# The Role of *Amh* in Gonadal Development and Its Response to Exogenous Hormones in Blotched Snakehead (*Channa maculata*)

**DOI:** 10.3390/ijms27198588

**Published:** 2026-09-25

**Authors:** Junqi Qin, Yueying Deng, Yuntao Lu, Yang Zhang, Luping Liu, Qing Luo, Haiyang Liu, Shuzhan Fei, Yuandong Sun, Jian Zhao, Mi Ou

**Affiliations:** 1 China (Guangxi)-ASEAN Key Laboratory of Comprehensive Exploitation and Utilization of Aquatic Germplasm Resources, Ministry of Agriculture and Rural Affairs, Key Laboratory of Aquaculture Genetic and Breeding and Healthy Aquaculture of Guangxi, Guangxi Academy of Fishery Sciences, Nanning 530021, China; 2 Key Laboratory of Tropical and Subtropical Fishery Resources Application and Cultivation, Ministry of Agriculture and Rural Affairs, Pearl River Fisheries Research Institute, Chinese Academy of Fishery Sciences, Guangzhou 510380, China; 3 School of Life and Health Sciences, Hunan University of Science and Technology, Xiangtan 411201, China

**Keywords:** Amh, gonadal development, male-biased expression, steroid hormone, gene editing

## Abstract

Anti-Müllerian hormone (Amh) is a key regulator of sex determination and differentiation in fish. In this study, we identified and characterized a single-copy *Amh* ortholog, designated *CmAmh*, in blotched snakehead (*Channa maculata*), an economically important species in China. The cDNA spans 2340 bp and encodes a 545-amino-acid protein with conserved AMH-N and TGF-β domains. *CmAmh* exhibited pronounced male-biased gonadal expression, with transcript levels in testes markedly exceeding those in ovaries across all developmental stages (45~365 days post-hatching (dph)). Cellular localization revealed *CmAmh* transcripts in the Sertoli cells and spermatogonia of the testis and, more weakly, in the oogonia and primary oocytes of the ovary, suggesting a primary role in spermatogenesis and a potential modulatory function in early oogenesis. A strong inverse correlation between promoter CpG methylation and transcriptional activity, hypermethylation in ovaries (82.5~89.7%) versus hypomethylation (15.8~19.1%) in testes, indicated epigenetic regulation, which functionally integrates with hormonal signaling: 17β-estradiol (E_2_) suppressed, whereas methyltestosterone (MT) upregulated *CmAmh* expression. Long-term E_2_ exposure induced male-to-female secondary sex reversal (SSR) in 26.7% of XY individuals, whereas MT failed to reverse XX fish, indicating that *CmAmh* modulation alone is insufficient to override ovarian fate. Furthermore, CRISPR/Cas9-mediated mutagenesis achieved 40~45% editing efficiency, predominantly generating frameshift alleles, providing a platform for future functional studies. Collectively, these findings position *CmAmh* as a hormone-responsive nodal point within a broader sex-differentiation network. This work provides a theoretical foundation for sex-control strategies in *C. maculata* aquaculture, while future studies should focus on homozygous mutant generation to definitively establish its loss-of-function phenotype and regulatory networks.

## 1. Introduction

Anti-Müllerian hormone (AMH), also known as Müllerian inhibiting substance (MIS), a member of the transforming growth factor-beta (TGF-β) superfamily, exerts evolutionarily conserved yet functionally diverse roles in vertebrate sex determination and differentiation [1]. During embryonic development, AMH drives Müllerian duct regression in male tetrapods, thereby directing the formation of the male reproductive tract while suppressing the female tract [2]. In adulthood, AMH expression persists in the gonads but exhibits sex- and cell-type-specific functions [3]. In males, AMH is secreted by Sertoli cells until puberty and inhibits spermatogenesis in Leydig cells [4]. In females, *Amh* expression is restricted to preantral and early antral follicles, where it critically regulates folliculogenesis by inhibiting primordial follicle recruitment [5]. The physiological significance of this ovarian function is underscored by loss-of-function evidence, as *Amh*-deficient mice display accelerated primordial follicle activation, an expanded pool of growing follicles, and consequent premature depletion of the ovarian reserve [6].

Despite the absence of Müllerian ducts in teleosts, *Amh* is highly conserved and plays diverse roles in sex determination and differentiation [7]. Notably, *Amh* or its Y-linked duplicate, *Amhy*, functions as the sex-determining gene (SDG) in 34 teleost species spanning multiple orders [8], including Atheriniformes (13) [9,10,11], Perciformes (2) [12,13], Esociformes (8) [14,15], Gasterosteiformes (4) [16,17,18], Scorpaeniformes (5) [19,20], Pleuronectiformes (1) [21], and Beloniformes (1) [22]. In most cases, this SDG originates from gene duplication, although allelic variation constitutes a rare alternative mechanism, as observed in Japanese flounder (*Paralichthys olivaceus*) and beloniform killifish (*Oryzias eversi*) [21,22]. Functional evidence strongly supports the essential role of Amhy in male sex determination: its knockdown or knockout in XY individuals of Patagonian pejerrey (*Odontesthes hatcheri*), Nile tilapia (*Oreochromis niloticus*), and *P. olivaceus* induces male-to-female sex reversal, whereas its overexpression in XX *O. niloticus* triggers female-to-male sex reversal [9,12,21].

Beyond this primary switching function, *Amh* expression exhibits marked species-specific patterns. In medaka (*Oryzias latipes*), *Amh* expression is sexually non-dimorphic, implying a role in gonadal maintenance rather than testicular specification [23]. In contrast, male-biased expression, consistent with a role in testicular differentiation, is observed in Japanese eel (*Anguilla japonica*) [24], northern snakehead (*C. argus*) [25], common carp (*Cyprinus carpio*) [26], and mandarin fish (*Siniperca chuatsi*) [27]. The association between Amh and testicular fate is further corroborated in sex-changing species. For example, in the protandrous black porgy (*Acanthopagrus Schlegeli*), *Amh* expression rises with testicular differentiation and declines upon male-to-female sex reversal [28]. In ricefield eel (*Monopterus albus*), its expression follows a male > intersex > female gradient and drives Sertoli cell differentiation to sustain male development and testicular function [29]. In the protogynous orange-spotted grouper (*Epinephelus coioides*), *Amh* overexpression upregulates male pathway genes (*Sox9*, *Dmrt1*) while suppressing female markers (*Cyp19a1a*, *Foxl2*) [30]. Collectively, these findings position Amh not only as a primary sex-determining switch but also as a critical downstream mediator of hormone-induced gonadal plasticity. This dual role is evidenced by its bidirectional transcriptional response to exogenous sex steroids. Treatments that reduce estrogen biosynthesis or increase androgen signaling, such as aromatase inhibitors (e.g., letrozole) or methyltestosterone (MT), consistently upregulate *Amh* expression and drive female-to-male sex reversal, as observed in *E. coioides* and *S. chuatsi* [31,32]. Conversely, estrogen administration suppresses *Amh* transcription and correlates with male-to-female sex reversal across multiple species, including *C. argus* [25], channel catfish (*Ictalurus punctatus*) [33], and *C. carpio* [34].

Promoter DNA methylation modulates *Amh* transcription during teleost sex differentiation, but its regulatory significance is species-specific. An inverse correlation, testicular hypomethylation coincident with elevated *Amh* expression, is observed in large yellow croaker (*Larimichthys crocea*) [35] and red crucian carp (*Carassius auratus* red var.) [36]. However, this pattern is not universal. In half-smooth tongue sole (*Cynoglossus semilaevis*) [37] and Asian seabass (*Lates calcarifer*) [38], testicular *Amh* expression persists despite promoter hypermethylation. Moreover, in *I. punctatus*, estrogen-induced transcriptional *Amh* suppression occurs without any discernible shift in methylation status [33]. These findings indicate that while promoter methylation may contribute to *Amh* regulation in certain lineages, it is neither universally required nor consistently predictive of transcriptional output, underscoring the lineage-specific complexity of epigenetic control in teleost sexual development.

Blotched snakehead (*Channa maculata*) is a commercially important freshwater fish valued for its culinary quality, high protein content, low intramuscular spine count, and medicinal properties [39]. Males exhibit superior growth performance compared to females, highlighting the economic potential of producing all-male populations through targeted sex-control strategies and necessitating elucidation of the underlying molecular mechanisms of sex determination and differentiation. Prior studies have shown that oral administration of 30 mg/kg 17β-estradiol (E_2_) for 40 days induces male-to-female sex reversal, and the resulting XY neofemales (XY-F) can be crossed with normal XY males (XY-M) to generate YY super-males (YY-M) [40]. Additionally, comparative transcriptomics has revealed that *Amh* displays significant sexual dimorphism, with markedly higher expression in testes than in ovaries [41]. In this study, we identified the *Amh* gene in *C. maculata* (designated *CmAmh*) and conducted comprehensive analyses of its expression across adult tissues and gonadal developmental stages, assessed its promoter methylation patterns in four gonadal tissue types, investigated its transcriptional responsiveness to exogenous hormone exposure (10 μg E_2_/MT per 1 g body weight), and generated CRISPR/Cas9-mediated mosaic mutants to investigate its functional role in sex differentiation and gonadal development. This work aims to clarify the regulatory and functional landscape of *CmAmh*, advance our understanding of sex differentiation mechanisms in *C. maculata*, and provide a theoretical foundation for the genetic breeding of all-male populations.

## 2. Results

### 2.1. Cloning and Sequence Analysis of CmAmh

The full-length *CmAmh* cDNA (GenBank accession no. PZ859211) contained a 186-bp 5′untranslated region (UTR), a 1638-bp open reading frame (ORF) encoding a 545-amino acid (aa) protein, and a 516-bp 3′UTR. The deduced protein included two conserved domains: an AMH N-terminal region (78~434 aa) and a TGF-β domain (452~545 aa) (Figure 1A and Figure S1).

Genomic DNA sequencing showed that *CmAmh* spans 8481 bp, with a 4539-bp 5′-flanking region, a 2801-bp coding sequence, and a 1141-bp 3′-flanking region. The gene contained seven exons and six introns, all flanked by canonical GT/AG splice sites (Figure 1B). Within the predicted promoter region (−1757~+1 bp), multiple putative TFBS were identified, including those for CCAAT/enhancer protein (C/EBP), GATA transcription factor (GATA-1), estrogen receptor (ER), Oct-1, Sp1, and activator protein 1 (AP-1) (Figure S2).

Multiple protein sequence alignments showed that *Cm*Amh shares high similarity with homologs from *C. argus* (96.33%) and *C. striata* (83.12%), but substantially lower identity with tetrapod orthologs, such as those of *Gallus gallus* (14.09%) and *Mus musculus* (14.05%) (Figure S3). Phylogenetic analysis placed *Cm*Amh within the teleost clade, distinct from tetrapods (Figure 2).

### 2.2. Expression Profiles of CmAmh in Adult Tissues and During Gonadal Development

In adult tissues, *CmAmh* expression exhibited pronounced sexual dimorphism in gonads, with significantly higher transcript levels in testes than in ovaries (*p* < 0.01, Figure 3A). Relative expression was 38.24 ± 2.96-fold in XY-M testes and 27.55 ± 2.39-fold in YY-M testes, compared with 0.94 ± 0.21-fold in XX-F ovaries and 2.22 ± 0.52-fold in XY-F ovaries. Outside the gonads, moderate expression was detected in muscle, liver, hypothalamus and pituitary, whereas low levels were observed in gills, spleen, intestines, brain, middle kidney, head kidney, and heart.

During gonadal development, testicular *CmAmh* expression consistently exceeded ovarian expression at all stages examined (*p* < 0.01, Figure 3B). In testes, expression was first detected at 45 dph (2.61 ± 0.04-fold), rose sharply to peak at 135 dph (133.75 ± 13.73-fold), and subsequently declined by 365 dph (42.65 ± 3.30-fold). In contrast, ovarian expression remained persistently low throughout development, ranging from a minimum at 75 dph (0.92 ± 0.12-fold) to a maximum at 135 dph (5.56 ± 0.22-fold). Across the entire 45- to 365-dph interval, testicular transcript abundance was 2.60- to 44.78-fold higher than that in the ovary (*p* < 0.01), indicating a male-biased expression pattern that emerges during early gonadal differentiation and is maintained thereafter.

### 2.3. DNA Methylation of the CmAmh Promoter

Bisulfite sequencing analysis of the *CmAmh* promoter revealed contrasting methylation patterns between ovarian and testicular gonadal phenotypes across the eight CpG sites examined (Figure 4). In ovarian tissues, both XX-F and XY-F samples exhibited significant hypermethylation, with mean methylation levels of 89.7 ± 6.5% and 82.5 ± 7.9%, respectively. However, the distribution of fully methylated sites differed between the two ovarian genotypes: while XX-F gonads displayed full methylation at six sites (2, 4, 5, 6, 7, 8) and partial methylation at sites 1 (20%) and 3 (93.3%) (Figure 4A), XY-F gonads showed full methylation only at sites 3 and 7, with the remaining six sites exhibiting high-level partial methylation (60.0~93.3%) and no site was completely unmethylated (Figure 4B). Conversely, both testicular genotypes (XY-M and YY-M) displayed globally low methylation levels (15.8 ± 5.7% and 19.1 ± 9.2%, respectively). Notably, site 7 was fully methylated in both XY-M and YY-M testes, while sites 2, 3, 5, 6, and 8 were completely unmethylated in both. Divergence between testicular genotypes was restricted to sites 1 and 4: XY-M testes showed complete demethylation at site 1 and 26.7% methylation at site 4 (Figure 4C), whereas YY-M testes exhibited 13.3% and 40% methylation at these respective sites (Figure 4D). Finally, a correlation analysis revealed a strong negative relationship between promoter CpG methylation levels and *CmAmh* transcript abundance (r = −0.9788, Figure S4), indicating that promoter hypermethylation is closely associated with transcriptional repression of *CmAmh*.

### 2.4. Cellular Location of CmAmh in Gonads

The spatial distribution of *CmAmh* transcripts was examined by ISH on gonadal sections from 135 dph XY-M and XX-F individuals, with adjacent HE-stained sections confirming typical gonadal histology. Ovarian sections from XX-F individuals contained oogonia (OG), primary oocytes (POC) and growing oocytes (GOC) (Figure 5A), while testicular sections from XY-M individuals contained spermatogonia (SG), primary spermatocytes (PSC) and secondary spermatocytes (SSC) (Figure 5B). In XX-F ovaries, weak hybridization signals were detected in OG and POC (Figure 5C). In XY-F testes, *CmAmh* transcripts were predominantly localized in Sertoli cells and SG, with weaker signals in PSC and SSC (Figure 5D). No specific signals were observed with the sense probe in either ovaries (Figure 5E) or testes (Figure 5F).

### 2.5. Expression Dynamics of CmAmh Following Exogenous Hormone Treatment

#### 2.5.1. Short-Term Hormone Treatment

The acute transcriptional response of *CmAmh* to exogenous E_2_ and MT was evaluated in gonads of 75 dph XX-F and XY-M individuals. Across all time points, control XY-M testes exhibited substantially higher basal *CmAmh* expression (0.75 ± 0.01~1.23 ± 0.10-fold) than control XX-F ovaries (0.02 ± 0.00~0.14 ± 0.03-fold), confirming the constitutive sexually dimorphic pattern established earlier (Figure 6A,B). In XX-F individuals, E_2_ treatment suppressed *CmAmh* expression, with the lowest level recorded at 48 h post-injection (hpi) (0.02 ± 0.00-fold). However, a transient and statistically significant elevation was observed at 72 hpi (0.21 ± 0.01-fold, *p* < 0.01 vs. XX-F controls), after which expression declined to below control levels by 144 hpi. By contrast, MT treatment induced a progressive increase in expression from 24 to 72 hpi, peaking at 72 hpi (0.33 ± 0.02-fold, *p* < 0.01 vs. XX-F controls), and remained elevated relative to controls for the remainder of the time course, despite moderate fluctuations thereafter (Figure 6A). In XY-M individuals, E_2_ treatment consistently suppressed *CmAmh* expression across all time points, with levels ranging from 0.11 ± 0.01 to 0.31 ± 0.03-fold, all significantly lower than those of XY-M controls (*p* < 0.01). Conversely, MT treatment elicited a robust upregulation beginning at 24 hpi, reaching a maximum at 72 hpi (3.99 ± 0.17-fold, *p* < 0.01 vs. XY-M controls), and remained significantly elevated relative to controls through the remaining sampling points (*p* < 0.01), albeit with a gradual decline after the peak (Figure 6B).

#### 2.5.2. Long-Term Hormone Treatment

To evaluate the sustained effects of hormonal manipulation, 75 dph XX-F and XY-M individuals received five biweekly injections over a 10-week period, with gonadal *CmAmh* expression quantified at 2, 4, 6, 8, and 10 weeks post-injection (wpi). During this period, E_2_ significantly suppressed *CmAmh* expression in XX-F individuals from 2 to 6 wpi relative to XX-F controls (*p* < 0.01), with levels returning to control values between 8 and 10 wpi. By contrast, MT treatment induced sustained upregulation in XX-F gonads, peaking at 10 wpi (1.14 ± 0.07-fold, *p* < 0.01 vs. XX-F controls) (Figure 6C). In XY-M individuals, E_2_ elicited a progressive decline in expression from 2 wpi onward, reaching its lowest level at 10 wpi (0.07 ± 0.00-fold), whereas MT induced a marked and persistent elevation, also peaking at 10 wpi (14.98 ± 1.14-fold, *p* < 0.01 vs. XY-M controls) (Figure 6D).

Four months after the final injection, gonadal phenotype and genetic sex were assessed in 30 randomly selected fish per group (Table 1). All control XX fish developed normal ovaries containing OG, POC, GOC, and mature oocytes (MOC) (designated CT-XX-O, Figure 7A). Long-term MT treatment of XX fish did not induce female-to-male sex reversal; their gonads retained ovarian structure but exhibited abundant scattered connective tissue (CT) (designated MT-XX-O, Figure 7B). In contrast, long-term E_2_ treatment of XX fish resulted in normal ovarian morphology (designated E_2_-XX-O, Figure 7C), similar to CT-XX-O. Conversely, E_2_ treatment of XY fish induced male-to-female sex reversal in 26.7% (4/15) of XY fish. Of these, two developed fully formed ovaries containing OG, POC, GOC, and MOC, accompanied by prominent somatic cell (SMC) proliferation (designated E_2_-XY-O, Figure 7D), while the other two developed ovotestes containing both oocytes and spermatocytes (designated E_2_-XY-OT, Figure 7E). By contrast, all control XY fish developed normal testes containing SG, PSC, SSC, spermatids (ST) and spermatozoa (SZ) (designated CT-XY-T, Figure 7F). MT-treated XY fish exhibited delayed spermatogenesis, with testes containing only SG, PSC, and SSC (designated MT-XY-T, Figure 7G). The remaining E_2_-treated XY fish maintained testicular structure (designated E_2_-XY-T, Figure 7H).

Endpoint quantification of *CmAmh* expression across these eight gonad types revealed a clear association with gonadal phenotype (Figure 8). Transcript levels were significantly higher in testes (CT-XY-T: 8.46 ± 0.47, MT-XY-T: 11.72 ± 1.21, and E_2_-XY-T: 5.55 ± 0.31-fold) than in ovaries (CT-XX-O: 1.00 ± 0.07, MT-XX-O: 1.27 ± 0.03, E_2_-XX-O: 0.59 ± 0.07 and E_2_-XY-O: 0.87 ± 0.04-fold), with no significant differences among the ovarian groups (*p* > 0.05). Notably, expression in the ovotestis (E_2_-XY-OT: 2.97 ± 0.08-fold) was intermediate between testicular and ovarian levels, further supporting a positive association between *CmAmh* expression and the degree of testicular differentiation.

### 2.6. Generation of CmAmh Mutants Using the CRISPR/Cas9 System

To generate targeted mutations in *CmAmh*, two gRNAs were designed to recognize distinct sites within Exons 3 and 4 of the cDNA (Figure 1B). Microinjection of the gRNA-Cas9 ribonucleoprotein complex yielded mutagenesis efficiencies of 40.0% for Amh-gRNA1 and 45.0% for Amh-gRNA2 (Table 2). Sequencing of alleles targeted by gRNA1 revealed four distinct mutation types: an 11-bp insertion (+11) and three deletions of 9, 7, and 5 bp (−9, −7, −5 bp) (Figure 9A). Among these, the −9 deletion resulted in an in-frame loss of 3 aa, while the +11, −7, and −5 mutations all introduced frameshifts that generated premature termination codons (Figure 9B). Similarly, Amh-gRNA2, targeting Exon 4, produced four deletion alleles (−153, −26, −10, and −8 bp) (Figure 9C), all of which also induced frameshifts and premature stop codons (Figure 9D). Thus, the designed gRNAs effectively cleaved the *CmAmh* locus, and the resulting frameshift-inducing indels are predicted to yield truncated, nonfunctional protein products.

## 3. Discussion

Amh is widely recognized as a key regulator of sex determination and gonadal differentiation across vertebrates. In this study, the identification of a single *Amh* copy in *C. maculata* contrasts with the presence of *Amhy* in several teleost lineages, including certain Perciformes (e.g., *O. niloticus*, *S. chuatsi*) [12,42], Scorpaeniformes (e.g., *Ophiodon elongatus*, *Sebastes schlegelii*) [19,20], and Atheriniformes (e.g., *O. hatcheri*, *O. argentinensis*) [9]. This copy-number variation highlights the evolutionary fluidity of the *Amh* locus, which has been independently co-opted as a sex-determining switch in distantly related fish groups [8]. However, the single-copy status observed in *C. maculata*, *C. argus* [25] and largemouth bass (*Micropterus salmoides*) [43] indicates that *Amh* duplication is not an obligate evolutionary pathway. In lineages lacking *Amhy*, sexual fate is likely governed by alternative genetic triggers, such as other master sex-determining genes (e.g., *Dmrt1*, *Gsdf*) [44], or by epigenetic and environmental factors that modulate downstream sex-differentiation genes [7]. Thus, while Amh is consistently implicated in testicular differentiation across teleosts, its genomic context and regulatory architecture vary considerably, reflecting the polygenic and labile nature of sex determination and differentiation in fish.

The seven-exon structure of *CmAmh*, in contrast to the five-exon arrangement typical of mammalian and avian *Amh*, is consistent with a conserved genomic architecture among teleosts, as observed in *C. argus* [25], *M. salmoides* [43], and *S. chuatsi* [42]. The presence of both AMH-N and TGF-β domains further indicates that the protein retains the structural features necessary for receptor binding and downstream signaling, suggesting functional conservation across vertebrates. Phylogenetic clustering of *Cm*Amh within the Perciformes clade supports the taxonomic classification of *C. maculata* and reinforces the evolutionary stability of Amh.

The pronounced testis-biased expression of *CmAmh* in adult gonads, with transcript levels significantly higher in testes than in ovaries, is consistent with the established paradigm of Amh as a male-associated factor across teleosts, as reported in *A. japonica* [24], *C. argus* [25], *C. carpio* [26], and *S. chuatsi* [27]. This sexual dimorphism emerges early during gonadal development, with testicular expression rising sharply from 45 to 135 dph and peaking coincident with the onset of active spermatogenesis, while ovarian expression remains persistently low throughout the developmental window examined. The timing of this peak coincides with the critical period of testicular differentiation and meiotic initiation [45], suggesting that *CmAmh* may play a role in establishing the spermatogenic environment. This temporal correlation is further supported by the localization of *CmAmh* transcripts to Sertoli cells, which provide structural and nutritional support for germ cells and secrete factors that regulate spermatogonial proliferation and meiosis [46]. The Sertoli-cell-specific expression of *CmAmh* is a conserved feature shared with *S. chuatsi* [27], *S. schlegelii* [20], *P. olivaceus* [47] and *E. coioides* [31]. These findings are consistent with the conserved role of Amh as a paracrine factor potentially involved in maintaining the spermatogonial niche and modulating germ cell progression in teleosts. In contrast to its prominent testicular expression, *CmAmh* was weakly expressed in the ovary, with transcripts detected in oogonia and primary oocytes. This pattern differs from that observed in *O. niloticus* [48], *E. coioides* [31] and *M. salmoides* [43], where *Amh* is localized to granulosa cells, but aligns with findings in *S. chuatsi* [27] and *S. schlegelii* [20], where expression occurs in oocytes. The functional significance of germ-cell-associated *Amh* expression in ovaries is not yet fully understood, but it may reflect a role in oocyte maturation or in the regulation of early folliculogenesis. In mammals, Amh is produced by granulosa cells of preantral and small antral follicles and acts to inhibit primordial follicle recruitment [49]. In teleosts, however, the ovarian function of Amh appears more variable and species-dependent. Given that *CmAmh* expression in the ovary is consistently lower than in the testis, its ovarian role is likely modulatory rather than determinative, and further studies are needed to clarify its contributions to female gametogenesis in *C. maculata*.

BSP revealed a robust inverse correlation between *CmAmh* promoter CpG methylation and transcript abundance: ovarian hypermethylation (82.5~89.7%) was associated with transcriptional silencing, whereas testicular hypomethylation (15.8~19.1%) correlated with high expression. This pattern is consistent with observations in *L. crocea* [35] and *C. auratus* [36], where *Amh* promoters are differentially methylated between sexes in an inversely correlated manner. These findings support a model in which DNA methylation serves as a repressive epigenetic mark that contributes to the sexually dimorphic expression of *Amh* in *C. maculata*, likely by modulating the accessibility of the promoter region to key transcription factors identified in the 5′-flanking region (e.g., GATA-1, ER, Sp1, AP-1). However, the relationship between *Amh* methylation and expression is not universally conserved across teleosts. In *C. semilaevis* [37] and *L. calcarifer* [38], *Amh* is strongly expressed in testes despite promoter hypermethylation, a finding that contradicts the expected inverse relationship. Such variability underscores the mechanistic heterogeneity of *Amh* regulation. Promoter methylation may be superseded by other regulatory layers in some lineages, such as histone modifications, chromatin remodeling, or the binding of transcriptional activators that override the repressive effects of methylation [50]. Alternatively, species-specific differences in promoter architecture, including the distribution and density of CpG sites, may influence the functional impact of methylation [51]. A further possibility is that Amh regulatory mechanisms evolved differently among species with distinct histories of *Amh* duplication. In *C. maculata*, no *Amhy* copy has been identified, and the observed association between promoter methylation and *CmAmh* expression suggests that epigenetic regulation may contribute to fine-tuning the single *Amh* copy in species lacking an *Amhy* duplicate. However, whether the absence of *Amhy* is specifically associated with methylation-mediated regulation of *Amh* remains unknown. Comparative analyses of *Amh* duplication status, promoter methylation, and *Amh* expression across teleost species are required to test this hypothesis. Thus, promoter methylation may represent one regulatory layer contributing to *Amh* transcription in *C. maculata*, although its relative contribution should be interpreted within the broader epigenetic and transcriptional regulatory network.

The bidirectional regulation of *CmAmh* by exogenous sex steroids, E_2_-induced suppression and MT-induced upregulation, demonstrates that *CmAmh* is integrated into the hormonal signaling network that governs gonadal fate. The rapid transcriptional responses observed within 24~72 hpi indicate that *CmAmh* is a direct or early-responding target of steroid signaling, likely mediated via conserved estrogen and androgen receptor elements identified in its promoter. The long-term hormone treatment data provide a functional link between *CmAmh* expression and gonadal phenotype. Notably, 26.7% of E_2_-treated XY individuals underwent male-to-female secondary sex reversal (SSR), with *CmAmh* expression reduced to ovarian-like levels, suggesting that repression of *CmAmh* is a critical step in the feminization process. This observation is consistent with findings in *O. niloticus*, where combined treatment with an androgen inhibitor and E_2_ for 60 days effectively induced male-to-female SSR in 30 dph XY fish, accompanied by the disappearance of *Cyp11b2* and *Dmrt1* in the ovaries of SSR individuals by 180 dph [52]. This proposition is further reinforced in juvenile zebrafish (*Danio rerio*), where estrogen signaling inhibits *Amh* and *Gsdf* expression during the critical window of sexual fate determination [53]. Furthermore, the intermediate *CmAmh* expression observed in E_2_-XY-OT supports a dose-dependent relationship between *Amh* transcript levels and the degree of testicular differentiation, suggesting that *CmAmh* may serve as a molecular metric of gonadal integrity. Conversely, in MT-treated XX individuals, despite sustained *CmAmh* upregulation, female-to-male SSR did not occur, indicating that *Amh* overexpression alone is insufficient to override ovarian determination in *C. maculata*. This contrasts with the efficacy of long-term aromatase inhibitor treatment, which induces female-to-male SSR, transforming morphologically differentiated ovaries into functional testes, in *D. rerio* [54], *O. latipes* [55], and *O. niloticus* [56], accompanied by downregulation of female-promoting genes and upregulation of male-promoting genes. These collective observations indicate that *CmAmh* functions as a responsive nodal point within the hormonal signaling cascade, but its modulation alone is insufficient to drive complete gonadal transdifferentiation. Rather, its expression may be associated with the activity of other key sex-determining and differentiation genes, such as *Dmrt1*, *Gsdf*, and *Cyp19a1a*, suggesting that Amh may participate in the regulatory network governing the balance between testicular and ovarian pathways.

The successful generation of CRISPR/Cas9-induced mosaic mutants, with mutation efficiencies of 40~45% and predominantly frameshift alleles, establishes a valuable platform for future loss-of-function studies in *C. maculata*. The predicted disruption of the TGF-β domain in most mutant alleles is expected to abrogate receptor binding and downstream signaling, rendering the protein nonfunctional. The functional consequences of *Amh* loss have been well documented across teleosts, revealing both conserved and species-specific phenotypes. In *D. rerio*, *Amh^−/−^* homozygous mutations increase the proportion of females and are accompanied by enhanced proliferation and abnormal differentiation of germ cells [57]. In *O. niloticus*, *Amh^+/−^* heterozygous mutations result in increased primary growth follicles and reduced fertility, while homozygous mutations lead to hypertrophic ovaries with failed transition from primary to vitellogenic follicles [48]. In *P. olivaceus*, *Amh*-mutant XY individuals exhibit complete male-to-female sex reversal, indicating that *Amh* signaling is indispensable for testicular differentiation [47]. Similarly, loss of the Y-linked *Amhy* results in complete gonadal sex reversal in XY mutants of *O. hatcheri* [9], *O. niloticus* [12], *S. chuatsi* [42], and *P. olivaceus* [21]. Collectively, these findings underscore the critical role of Amh in male sex determination and differentiation, gonadal development, and early germ cell regulation in fish. Due to the young age of the founder mutants generated in this study, we are currently unable to assess whether *CmAmh* loss affects gonad development, spermatogenesis, or the expression of related genes in *C. maculata*. The production of homozygous *CmAmh^−/−^* mutants will require two consecutive generations of breeding, and we plan to pursue this in future investigations.

## 4. Materials and Methods

### 4.1. Experimental Fish and Sample Collection

Experimental blotched snakeheads were reared at the Fangcun Experiment Station of Pearl River Fisheries Research Institute (Guangzhou, China). For adult tissue distribution analysis, one-year-old individuals from four genotypic-phenotypic groups: normal XX females (XX-F), XY-M, XY-F, and YY-M, were identified using sex-specific molecular markers combined with histological gonadal examination, following established protocols [58]. Prior to dissection, fish were anesthetized with 1.25 g/L MS-222 (Sigma-Aldrich, Burlington, MA, USA). From each group, tissue samples, including middle kidney (MK), spleen (S), gills (G), muscle (M), liver (L), gonad (ovary (O)/testis (T)), pituitary (P), head kidney (HK), intestines (I), brain (B), heart (H), and hypothalamus (Hy), were collected (*n* = 3 per group), immediately flash-frozen in liquid nitrogen, and stored at −80 °C.

To assess the ontogenetic expression profile of *CmAmh*, gonads were separately collected from XX-F and XY-M individuals at seven developmental stages: 45, 75, 105, 135, 165, 195, and 365 days post-hatching (dph; *n* = 3 per sex per stage). Genetic sex was confirmed as previously described [58]. All gonadal samples were flash-frozen in liquid nitrogen and stored at −80 °C until further processing. All animal procedures complied with institutional welfare guidelines and were approved by the Animal Ethics Committee of the Pearl River Fisheries Research Institute, Chinese Academy of Fishery Sciences.

### 4.2. Hormone Treatment

A cohort of 720 blotched snakeheads (75 dph, body weight: 58.9 ± 3.1 g, body length: 14.5 ± 0.3 cm) was randomly allocated to six treatment groups (120 fish per group), each distributed across three replicate concrete ponds (2 m × 2 m × 1 m; 40 fish per pond). E_2_ and MT (Aladdin, Shanghai, China) were dissolved in corn oil and administered via intraperitoneal (i.p.) injection. For the short-term regimen (Groups 1–3), fish received a single i.p. injection of either 10 µg/g body weight E_2_ (Group 1), 10 µg/g body weight MT (Group 2), or an equivalent volume of corn oil vehicle (Control; Group 3). Gonads were collected from both XX-F and XY-M individuals (*n* = 3 per sex per time point) at 24, 48, 72, 96, 120, and 144 hpi. For the long-term regimen (Groups 4–6), fish received five i.p. injections at two-week intervals over a 10-week period, with dosages and protocols identical to those described above. Gonads were collected from XX-F and XY-M individuals (*n* = 3 per sex per time point) at 2, 4, 6, 8, 10 wpi. Genetic sex was confirmed for all sampled individuals following previously established methods [59]. All gonadal samples were flash-frozen in liquid nitrogen and stored at −80 °C until further processing.

Throughout the experiment, fish were maintained under identical environmental conditions and fed a commercial compound feed (45% crude protein) twice daily to apparent satiation (8~10% body weight). Water quality parameters were kept within optimal aquaculture ranges: temperature 26~28 °C, dissolved oxygen 6~8 mg/L, ammonia nitrogen < 0.5 mg/L, nitrite < 0.01 mg/L, and pH 7.0~7.5, with a 20% water exchange performed every three days.

### 4.3. Gene Cloning and Sequence Analysis of CmAmh

The cDNA and genomic DNA (gDNA) sequences of *CmAmh* were predicted from the blotched snakehead genome (SRA Accession No. PRJNA730430) [58]. RNA isolation, cDNA synthesis, and gene-specific PCR amplification using gene-specific primers (primers listed in Table 3) were performed following established protocols [59], and the products were sequenced for confirmation. For comparative analysis, Amh protein sequences from 19 vertebrate species, including *Channa argus*, *Channa striata*, *Siniperca chuatsi*, *Micropterus salmoides*, *Lates calcarifer*, *Lates japonicus*, *Oreochromis niloticus*, *Cyprinus carpio*, *Danio rerio*, *Cynoglossus semilaevis*, *Pelodiscus sinensis*, *Xenopus tropicalis*, *Geotrypetes seraphini*, *Gallus gallus*, *Columba livia*, *Mus musculus*, *Bos taurus*, *Equus caballus* and *Homo sapiens*, were retrieved from NCBI (https://www.ncbi.nlm.nih.gov, accessed on 16 February 2026). Multiple sequence alignment was conducted with DNAMAN, and a neighbor-joining (NJ) phylogenetic tree (1000 bootstrap replicates) was constructed using MEGA 11.0. Conserved domain architecture was examined using the Simple Modular Architecture Research Tool (SMART) (http://smart.embl-heidelberg.de/, accessed on 14 January 2026).

To determine the genomic structure, gDNA was extracted from tail tissue using a Universal Genomic DNA Kit (CWBIO, Taizhou, China), and the *CmAmh* genomic region was amplified with specific primers (Table 4) designed from the predicted genome sequence [58]. Exon–intron boundaries were determined by aligning gDNA and cDNA sequences. The promoter core element (PCE) and transcript start site (TSS) were predicted by NNPP (http://www.fruitfly.org/seq_tools/promoter.html, accessed on 16 January 2026), and potential transcription factor binding sites were identified via AliBaba 2.1 (http://generegulation.com/pub/programs/alibaba2/index.html, accessed on 16 January 2026) and Animal TFDB (http://bioinfo.life.hust.edu.cn/AnimalTFDB/, accessed on 16 January 2026).

### 4.4. Quantitative Real-Time PCR (qRT-PCR)

qRT-PCR was performed in triplicate using SYBR^®^ Green Master Mix (Toyobo, Osaka, Japan) on a StepOnePlus™ Real-Time PCR System (Applied Biosystems, Foster City, CA, USA), following the manufacturer′s instructions, with the primer pairs listed in Table 3. Each 20 μL reaction contained 10 μL SYBR^®^ Green Master Mix, 0.8 μL each of forward and reverse primers (10 μM), 7.4 μL ddH_2_O, and 1 μL cDNA. Expression levels were normalized to the housekeeping genes β-actin and EF1α [59] and relative quantification was performed using the 2^−∆∆Ct^ method. To enable appropriate experimental comparisons, distinct calibrator samples (defined as expression = 1.0) were employed across different series: gills of XX-F adults for tissue distribution, ovaries of XX-F at 45 dph for gonadal development, and ovaries of control XX-F (designated CT-XX-O) for the long-term hormone induction. For short-term hormone induction, relative expression levels of *CmAmh* were calculated using the 2^∆Ct^ method relative to the internal housekeeping genes.

### 4.5. Bisulfite Sequencing PCR (BSP)

Genomic DNA was extracted from gonads of XY-M, YY-M, XX-F, and XY-F individuals (*n* = 3 per group), and equal amounts of DNA from the three biological replicates were pooled per group. Bisulfite conversion was performed using the EZ DNA Methylation-Gold™ Kit (Zymo Research, Irvine, CA, USA). A CpG island within the *CmAmh* promoter was predicted with MethPrimer (http://www.urogene.org/cgi-bin/methprimer/methprimer.cgi, accessed on 17 January 2026), and gene-specific primers were designed (Table 3). Following PCR amplification, products were cloned and sequenced. For each group, 15 clones (5 per individual) were randomly selected, and the CpG methylation percentage was calculated as: (number of methylated CpGs/total CpGs analyzed) × 100% [59].

### 4.6. In Situ Hybridization (ISH)

Gonads from 135 dph XX-F and XY-M individuals were fixed in Bouin’s solution for hematoxylin and eosin (HE) staining and ISH. Gene-specific probes for *CmAmh* were generated by PCR using primers listed in Table 3. The resulting amplicons were purified, sequenced, and verified by DNAMAN (version 6.0.3.99) alignment against the blotched snakehead reference genome. HE staining, probe synthesis, and ISH were carried out in accordance with our previous research [60].

### 4.7. CRISPR/Cas9-Mediated CmAmh Knockout

Two guide RNAs (gRNAs) targeting the *CmAmh* locus (Table 5) were designed using CRISPRscan (https://www.crisprscan.org/, accessed on 20 January 2026) and synthesized with the MAXIscript T7 In Vitro Transcription Kit (Invitrogen, Carlsbad, CA, USA), followed by LiCl/ethanol purification. A microinjection mixture containing 200 pg of gRNA and 300 pg TrueCut™ Cas9 protein v2 (Invitrogen, Carlsbad, CA, USA) was injected into embryos at the 1~4 cell stage. Mutagenesis efficiency and editing fidelity were confirmed by Sanger sequencing of 20 randomly selected embryos per group. Artificial insemination, microinjection, and subsequent larval rearing followed established protocols [61].

### 4.8. Statistical Analysis

All data are presented as means ± standard deviation (SD). Differences among experimental groups were evaluated using one-way analysis of variance (ANOVA) followed by Dunnett’s post hoc test (SPSS 26.0, IBM, Chicago, IL, USA), with statistical significance set at *p* < 0.05. The correlation between *CmAmh* promoter CpG methylation levels and corresponding transcript abundances was assessed using Pearson’s correlation coefficient, calculated in Microsoft Excel 2021.

## 5. Conclusions

In this study, we identified and characterized *CmAmh*, a single-copy *Amh* ortholog in *C. maculata*, which exhibits a conserved seven-exon structure and AMH-N and TGF-β domains. *CmAmh* displayed pronounced male-biased gonadal expression, with testicular transcript levels far exceeding ovarian levels across all developmental stages. *CmAmh* transcripts localize to Sertoli cells and, more weakly, to oogonia and primary oocytes, suggesting a primary role in spermatogenesis and a potential modulatory function in early oogenesis. A strong inverse correlation between promoter CpG methylation and transcriptional activity, hypermethylation in ovaries versus hypomethylation in testes, indicates epigenetic regulation, which is functionally integrated with hormonal signaling: E_2_ suppresses while MT upregulates *CmAmh* expression. Notably, long-term E_2_ exposure induced male-to-female SSR in 26.7% of XY individuals, whereas MT failed to reverse XX fish, indicating that *CmAmh* modulation alone is insufficient to override ovarian fate. Furthermore, the successful generation of CRISPR/Cas9-induced mosaic mutants, with mutation efficiencies of 40~45% and predominantly frameshift alleles, establishes a valuable platform for future loss-of-function studies in *C. maculata*. The responsiveness of *CmAmh* to exogenous steroids and its amenability to CRISPR/Cas9 disruption offer promising avenues for sex-control strategies in aquaculture. Future work should focus on generating homozygous *CmAmh^−/−^* mutants to definitively establish its loss-of-function phenotype and to delineate its upstream and downstream regulatory networks.

## Figures and Tables

**Figure 1:**
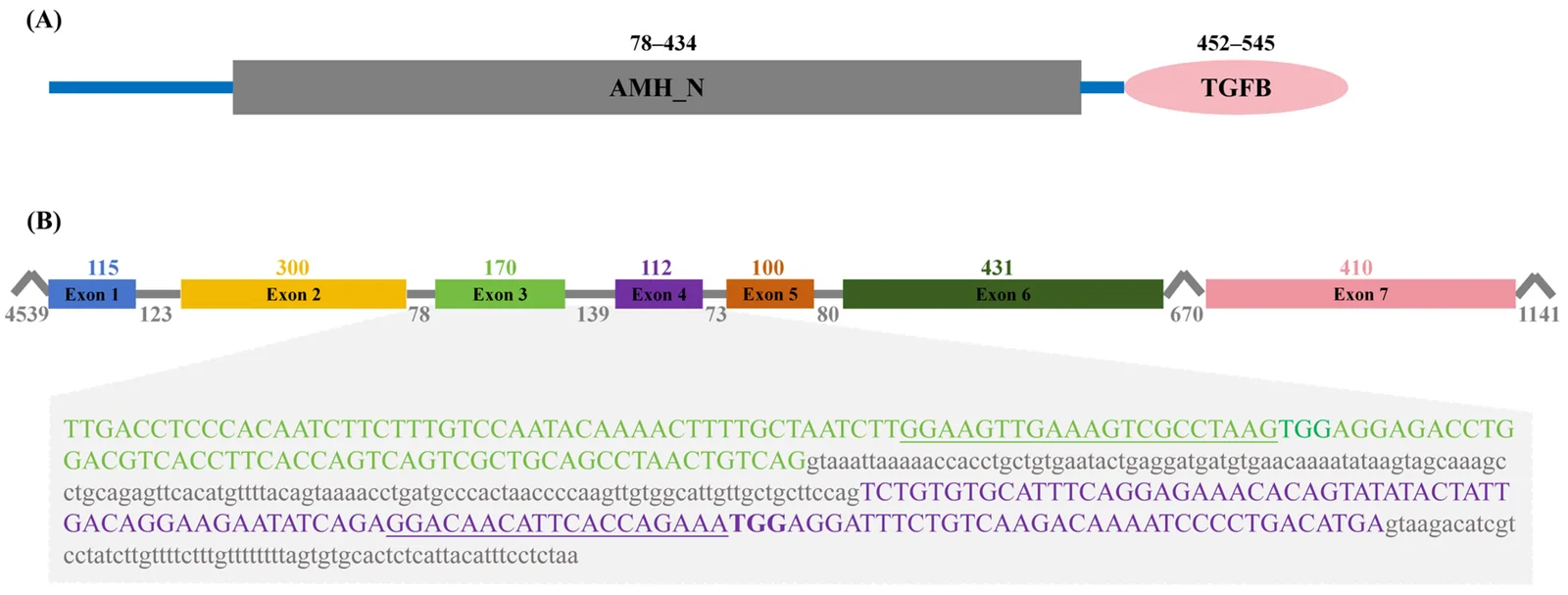
Schematic diagram of *Cm*Amh. (**A**) Predicted protein structure. (**B**) Genomic structure. Boxes represent exons; introns are shown as intervening lines. Numbers above and below indicate exon and intron lengths (bp), respectively. CRISPR/Cas9 target sites are located in Exons 3 and 4. Exons and introns are denoted by uppercase and lowercase letters, respectively. gRNA target sites are indicated by underscores; the PAM sequence is in bold.

**Figure 2:**
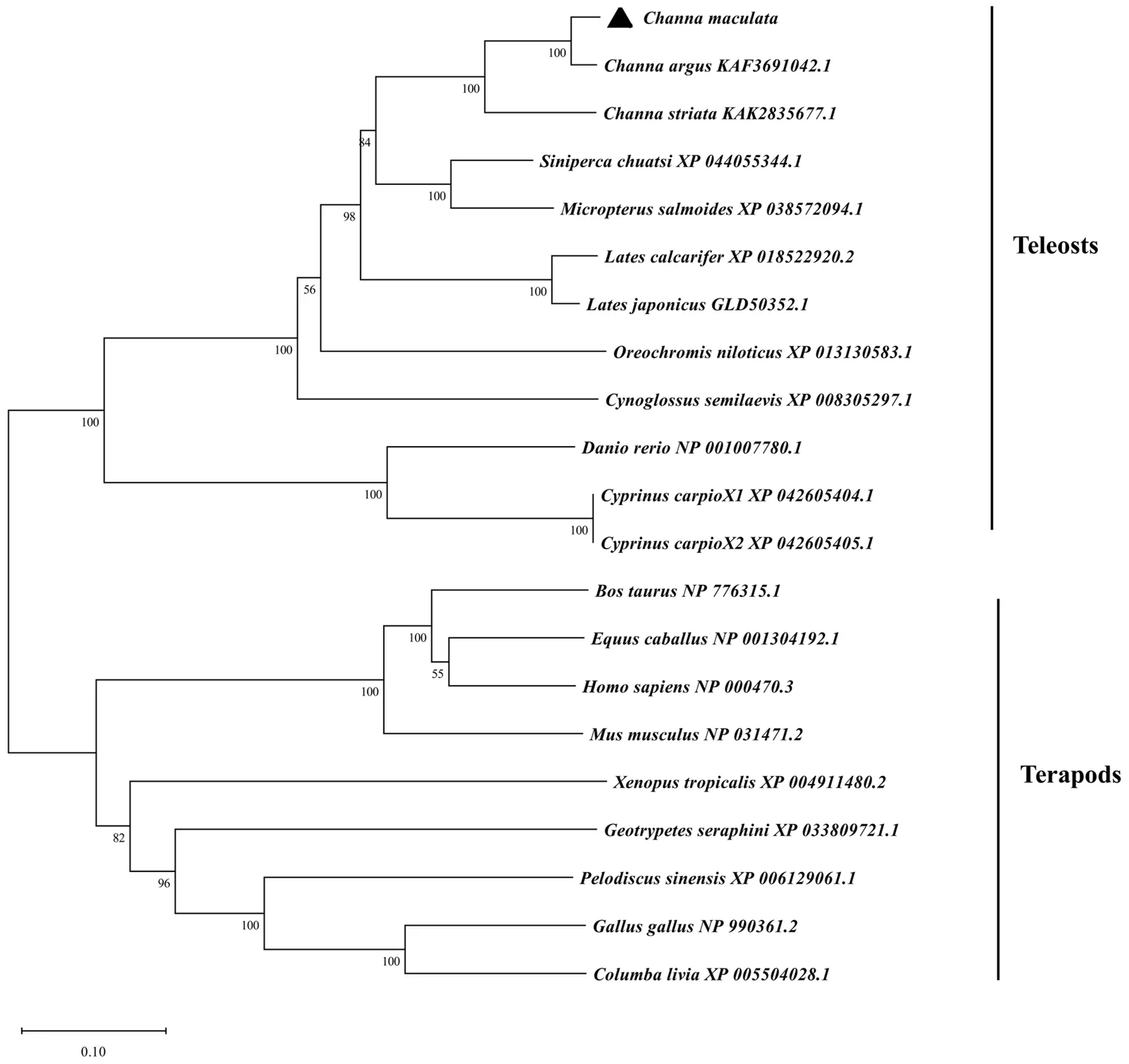
The NJ phylogenetic tree based on Amh amino acid sequences from various vertebrate species. Bootstrap values over 30% are shown. The scale bar represents genetic distance. *Cm*Amh is marked with a triangle.

**Figure 3:**
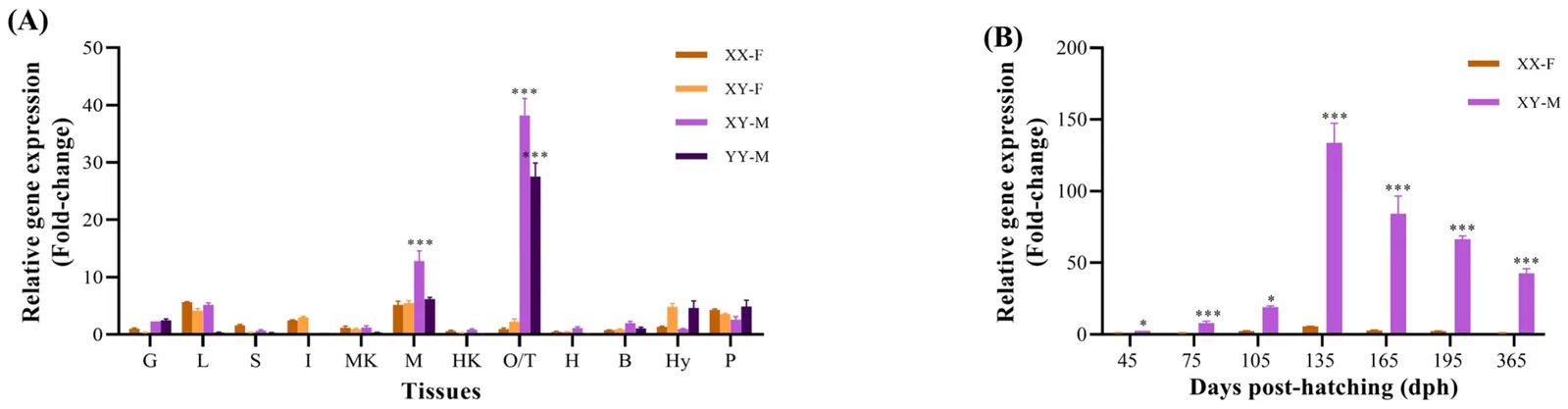
Expression patterns of *CmAmh* transcripts. (**A**) Tissue-specific expression in four types of *C. maculata*, normalized to expression in gills (G) of XX-F adults. G: gills, L: liver, S: spleen, I: intestines, MK: middle kidney, M: muscle, HK: head kidney, O/T: ovary/testis, H: heart, B: brain, Hy: hypothalamus, P: pituitary. (**B**) Relative expression in gonads of XX-F and XY-M individuals at different developmental stages, normalized to expression in XX-F ovaries at 45 dph. *β-actin* and *EF1α* were used as reference genes. Data are mean ± SD (*n* = 3). “*”: *p* < 0.05, “***”: *p* < 0.001.

**Figure 4:**
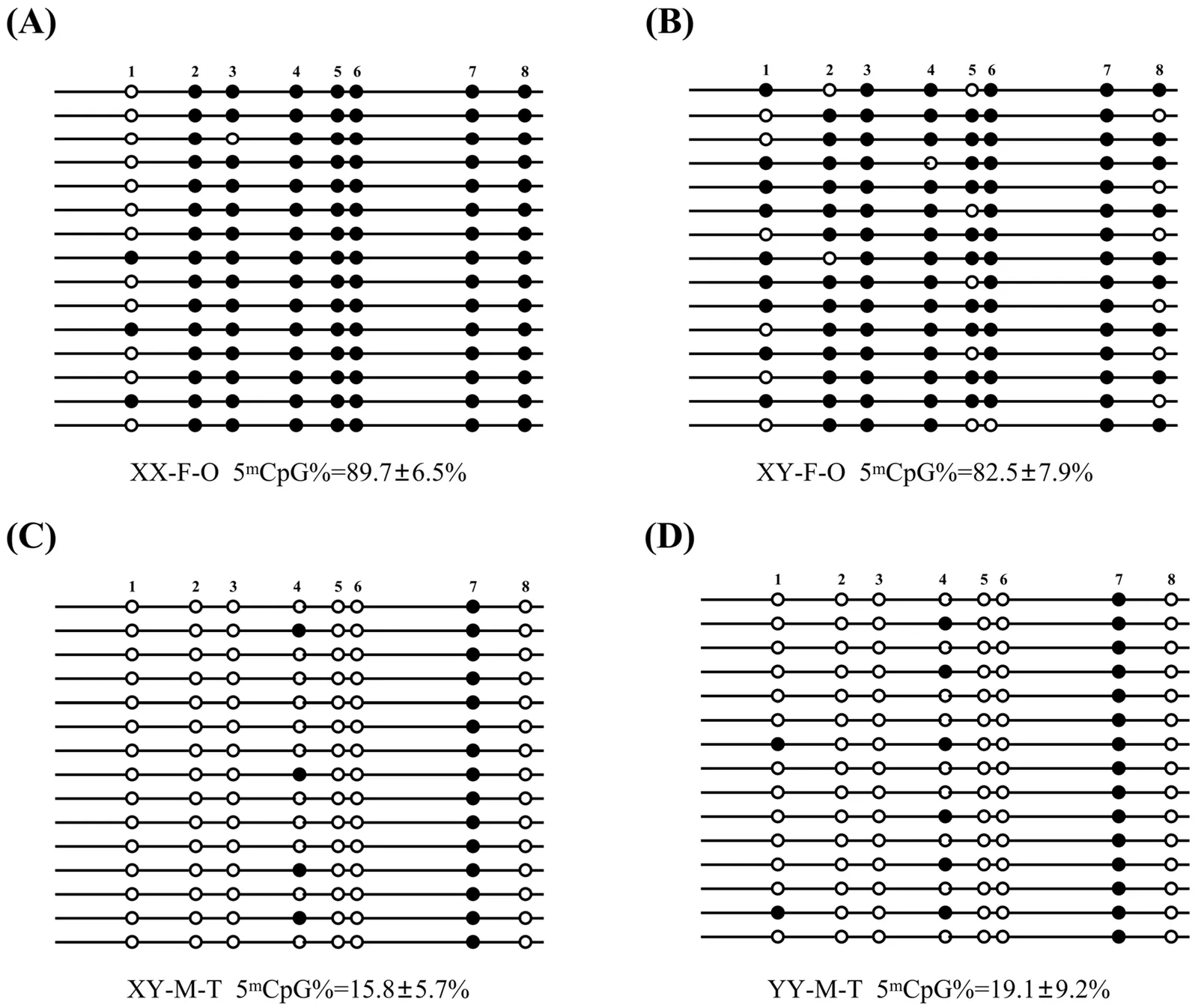
CpG methylation profiles of the *CmAmh* promoter in gonads of four *C. maculata* types. (**A**) XX-F. (**B**) XY-F. (**C**) XY-M. (**D**) YY-M. The numbers 1–8 indicate eight different CpG sites. Filled circles indicate methylated CpG sites; open circles indicate unmethylated CpG sites. Values are mean ± SD (*n* = 15).

**Figure 5:**
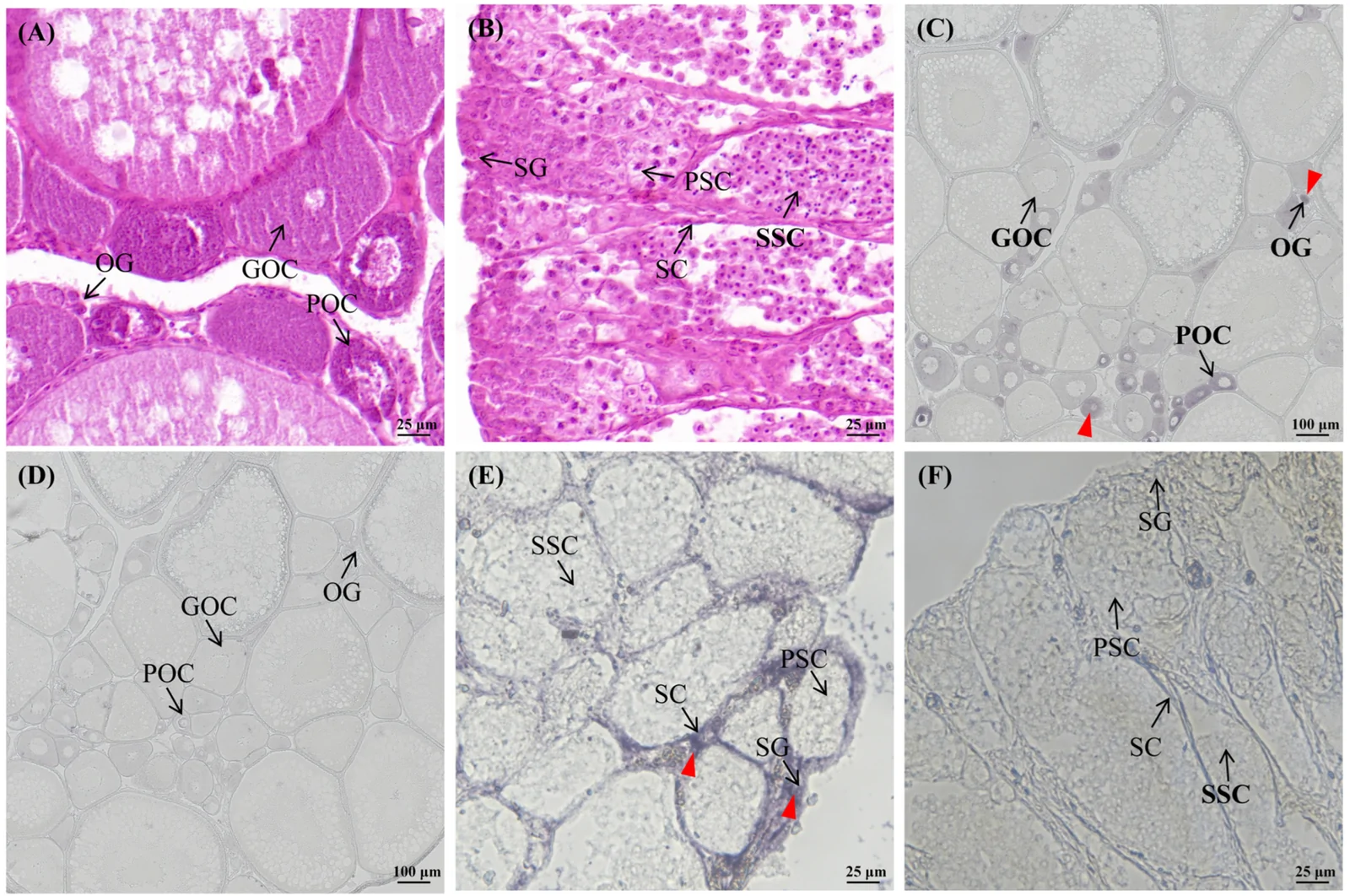
Cellular localization of *CmAmh* mRNA in XX-F ovaries and XY-M testes. (**A**,**B**) HE staining of the ovary and testis, respectively. (**C**,**E**) ISH with antisense probes in the ovary and testis, positive signals are blue. (**D**,**F**) Negative controls with sense probes in the ovary and testis, respectively. Positive signals are indicated by red arrows. OG: oogonia, POC: primary oocytes, GOC: growing oocytes, SG: spermatogonia, PSC: primary spermatocytes, SSC: secondary spermatocytes, SC: Sertoli cell.

**Figure 6:**
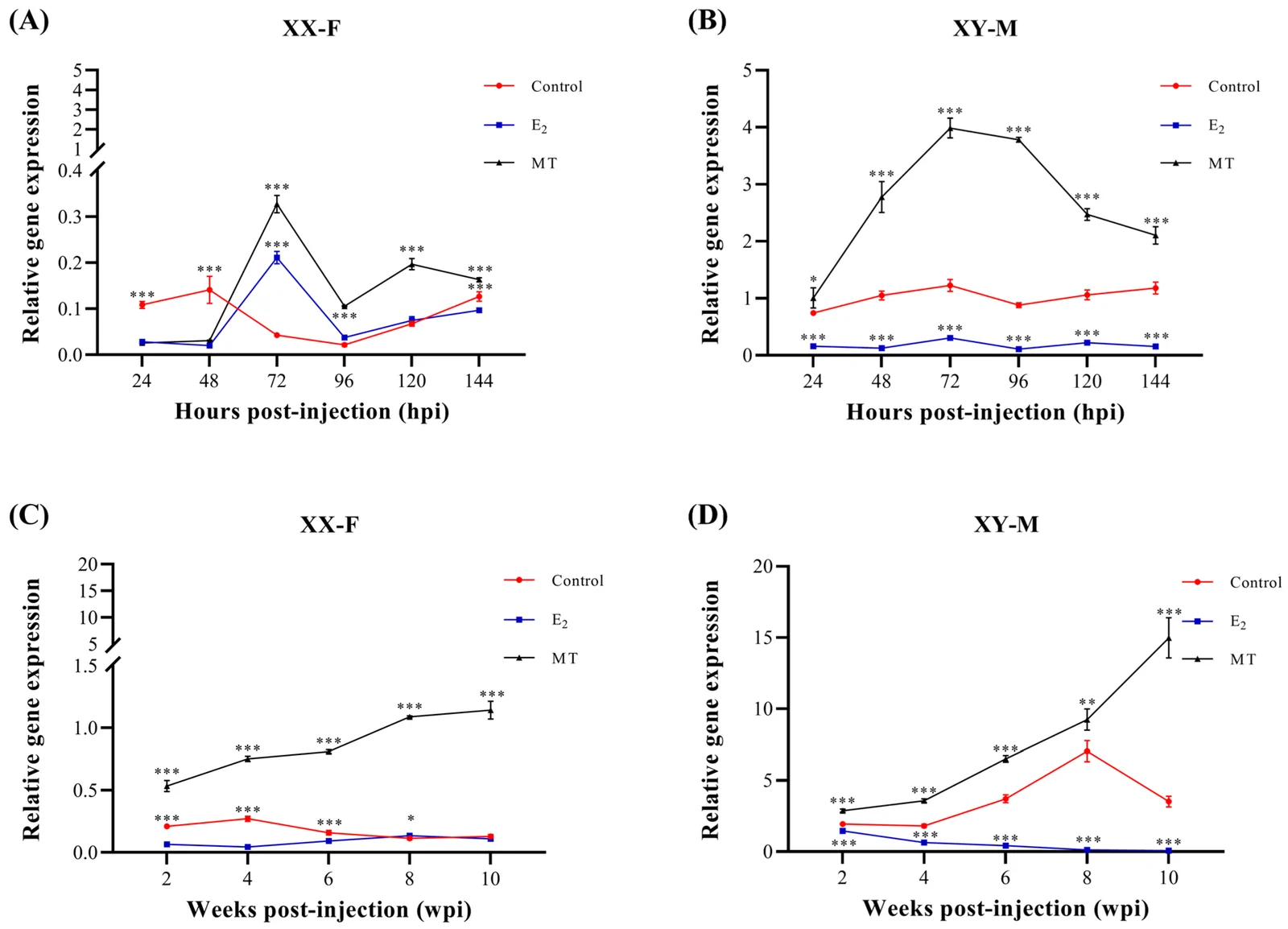
Expression dynamics of *CmAmh* in gonads of XX-F and XY-M individuals following E_2_ and MT treatment (*n* = 3). (**A**,**B**) Short-term treatment in XX-F and XY-M individuals, respectively. (**C**,**D**) Long-term treatment in XX-F and XY-M individuals, respectively. Data are mean ± SD (*n* = 3). “*”: *p* < 0.05, “**”: *p* < 0.01, “***”: *p* < 0.001.

**Figure 7:**
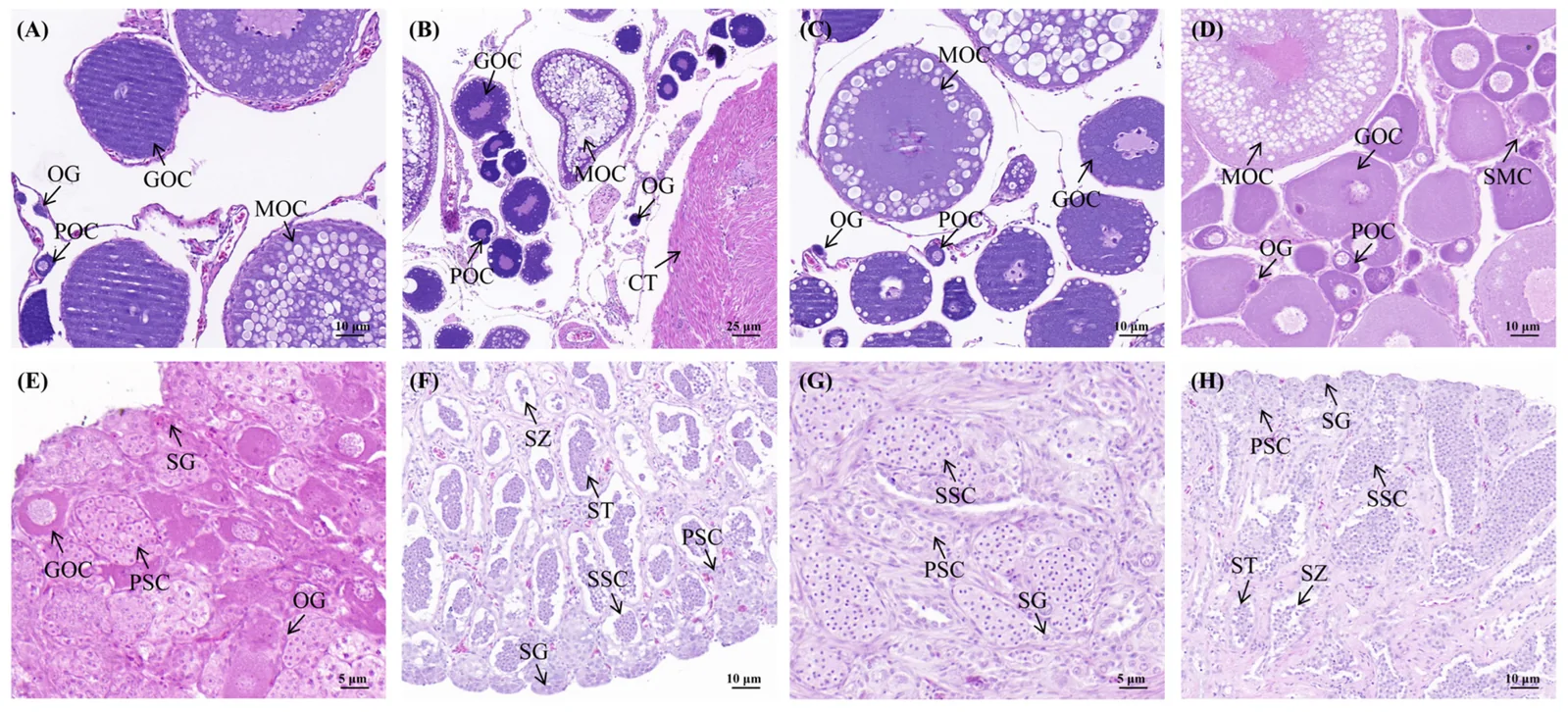
Histological observations of gonads from control and long-term hormone-treated fish. (**A**) Control XX ovary (CT-XX-O). (**B**) MT-treated XX ovary (MT-XX-O). (**C**) E_2_-treated XX ovary (E_2_-XX-O). (**D**) E_2_-treated XY ovary (E_2_-XY-O). (**E**) E_2_-treated XY ovotestis (E_2_-XY-OT). (**F**) Control XY testis (CT-XY-T). (**G**) MT-treated XY testis (MT-XY-T). (**H**) E_2_-treated XY testis (E_2_-XY-T). OG: oogonia, POC: primary oocytes, GOC: growing oocytes, MOC: mature oocytes, SG: spermatogonia, PSC: primary spermatocytes, SSC: secondary spermatocytes, ST: spermatids, SZ: spermatozoa, CT: connective tissue, SMC: somatic cell.

**Figure 8:**
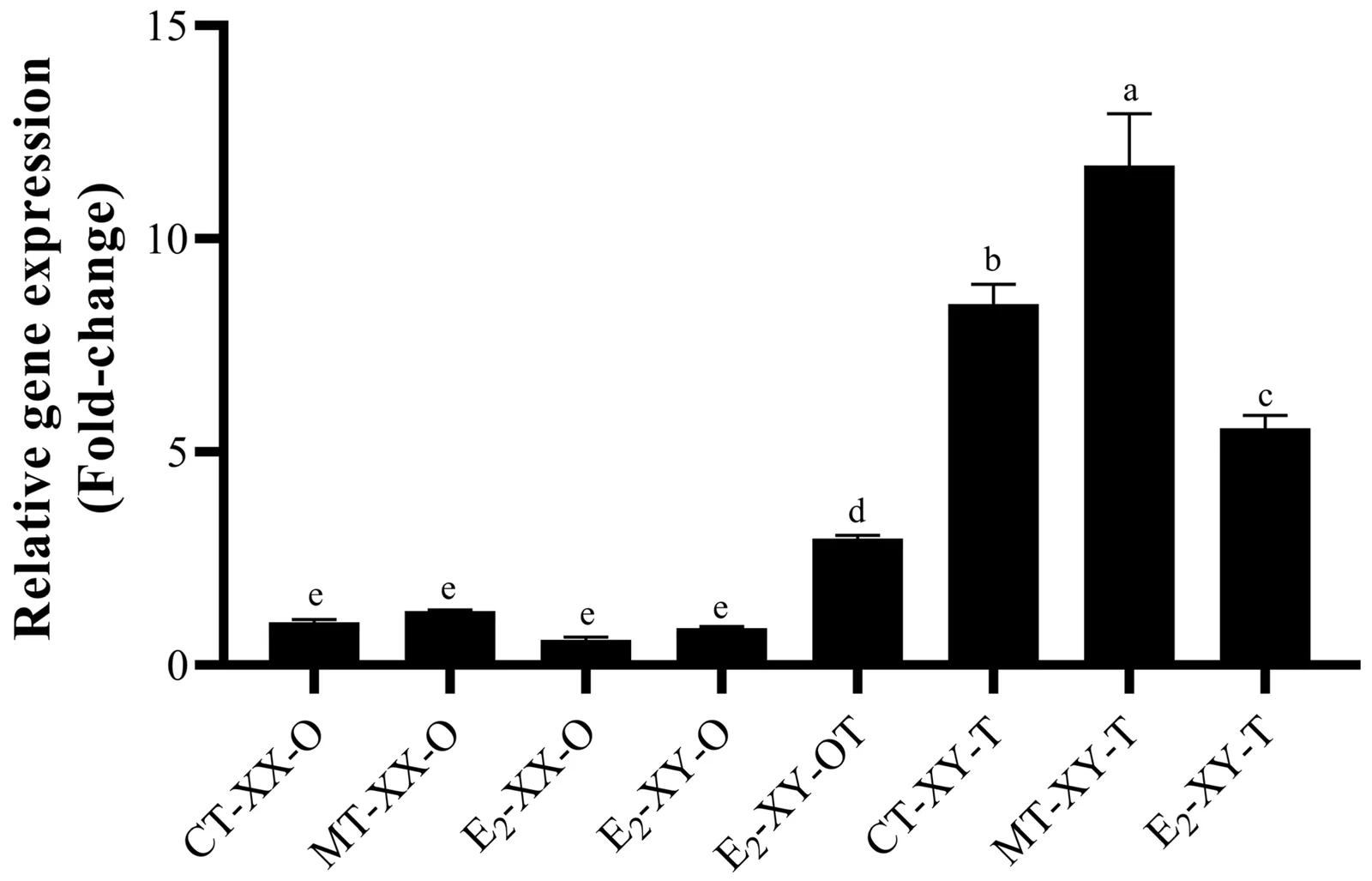
Expression profiles of *CmAmh* in eight gonad types from control and long-term hormone-treated fish. CT-XX-O: control XX ovary, MT-XX-O: MT-treated XX ovary, E_2_-XX-O: E_2_-treated XX ovary, E_2_-XY-O: E_2_-treated XY ovary, E_2_-XY-OT: E_2_-treated XY ovotestis, CT-XY-T: control XY testis, MT-XY-T: MT-treated XY testis, E_2_-XY-T: E_2_-treated XY testis. The different letters above the error bars indicate a significant difference (*p* < 0.05). Values are mean ± SD (*n* = 3).

**Figure 9:**
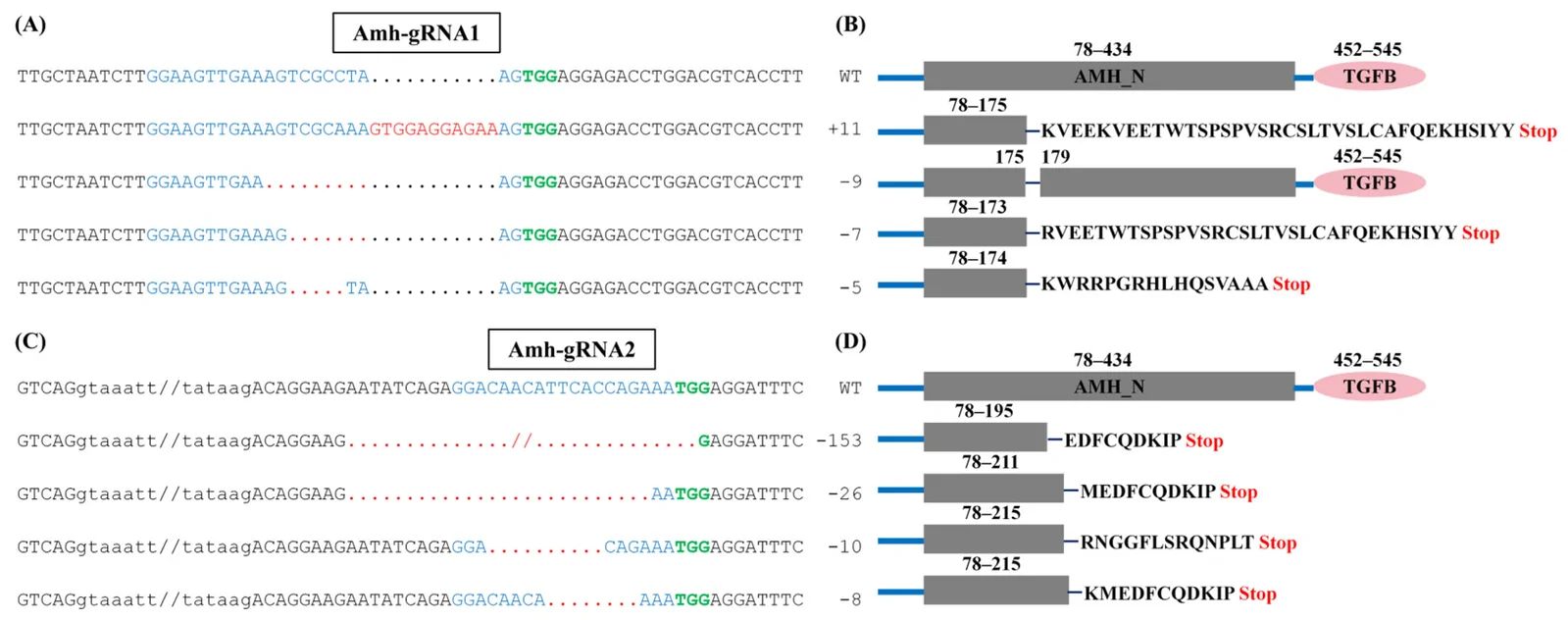
CRISPR/Cas9-mediated modifications of *CmAmh*. (**A**,**C**) Sequences showing mutations induced by co-delivered gRNA and Cas9 protein. Wild-type (WT) sequences are shown at the top; the gRNA target site is in blue, followed by the PAM sequence (in bold green). Deletions (−) and insertions (+) are indicated, with numbers to the right showing the change in base pairs. Indels of base pairs are in red. (**B**,**D**) Schematic diagrams of predicted intact WT Amh protein and truncated protein in mosaic F0 *C.maculata*. Numbers indicate amino acid residue positions. Frameshifts generate altered amino acid sequences after the incomplete domain, leading to a premature termination codon (in bold red).

**Table 1:** Genetic sex and gonadal phenotype of control and long-term hormone-treatment fish.

Group	Genetic Sex	Fish Examined	Testis	Ovotestis	Ovary	Sex Reversal Rate (%)
Control	XX	14	0	0	14	0
	XY	16	16	0	0	0
E_2_	XX	15	0	0	15	0
	XY	15	11	2	2	26.7
MT	XX	12	0	0	12	0
	XY	18	18	0	0	0

**Table 2:** Mutation types of *CmAmh* detected by PCR amplification, subcloning and sequencing in mosaic F0 blotched snakehead.

gRNA	Mutation Type	Number of Clones Carrying Mutated *Amh*	Mutation Rate
Amh-gRNA1	WT	12	40.0%
	+11	2	
	−9	1	
	−7	2	
	−5	3	
Amh-gRNA2	WT	11	45.0%
	−153	3	
	−26	3	
	−10	2	
	−8	1	

Note: Deletions are denoted by “−”, insertions by “+”. Mutation rate (%) = (number of clones carrying mutated *CmAmh*)/(total clones examined) × 100%.

**Table 3:** Primers used for full-length cDNA cloning, qRT-PCR, BSP, ISH and knockout detection.

Primer Name	Sequence (5′→3′)	Application
Amh-F1	GATGACCTATTTGCAGCG	Partial sequence obtaining
Amh-R1	GTTGAGCAGAACAGCGTG	
Amh-5′ Rout	CTCCACTTAGGCGACTTTCAACTTCC	5′-Race PCR amplification
Amh-5′ Rin	GCTTGATGAATTTTCAGATGCTGTGC	
Amh-3′ Fout	AGACCCCAACAATCCAGTGAGAAGC	3′-Race PCR amplification
Amh-3′ F in	TGGCTCTTGTACATTCCCTCTGGTC	
Amh-F2	AACAGGCGATTGAGAAAGGT	ORF qualifying
Amh-R2	AGACCTTCAGCGGCATCC	
Amh-BSP-F1	TCAGATATTGTAGTGAAATAGATCT	BSP
Amh-BSP-R1	ATCAGACCTTTCTCAATCG	
Amh-BSP-F2	TTAGATATTGTAGTGAAATAGATTT	
Amh-BSP-R2	ATCAAACCTTTCTCAATCG	
Amh-qF	ATAGAGAGGCTGGGGAGACTCAA	qRT-PCR
Amh-qR	TCTCACTGGATTGTTGGGGTCT	
β-actin-qF	GCAAGCAGGAGTATGATGAG	
β-actin-qR	TTGGGATTGTTTCAGTCAGT	
EF1α-qF	GGGACACCCACAATAACATCC	
EF1α-qR	CCAGGCATACTTGAAGGAGC	
Amh-ISH-F	AATCGGCTTATCCTCCAGCG	ISH
Amh-ISH-R	GGACCCACAACGTGCTTTTC	
Amh-ISH-T7-zy-F	TAATACGACTCACTATAGGGAATCGGCTTATCCTCCAGCG	
Amh-ISH-T7-zy-R	GGACCCACAACGTGCTTTTC	
Amh-ISH-T7-fy-F	AATCGGCTTATCCTCCAGCG	
Amh-ISH-T7-fy-R	TAATACGACTCACTATAGGGGGACCCACAACGTGCTTTTC	
Amh-JC-F	TTGACCTCCCACAATCTTCTT	Knockout detection
Amh-JC-R	TTAGAGGAAATGTAATGAGAGTGC	

**Table 4:** Primers used for genomic DNA cloning.

Primer Name	Sequence (5′→3′)	Length (bp)
Amh-gDNA-F1	GGGATGTTGGTCAGAGAGTGTA	1300
Amh-gDNA-R1	TCAACAATAATCACAAAAGACCTC	
Amh-gDNA-F2	AATGCCGATGTAAAATACTTGTG	1915
Amh-gDNA-R2	TGGACAACTCCTATTTCTCTTCTT	
Amh-gDNA-F3	TGAGAACAAACAGATGTCAAAGC	1782
Amh-gDNA-R3	GTGGATTCTTTTTGAGATGTTTG	
Amh-gDNA-F4	GCAAACATCTCAAAAAGAATCCAC	919
Amh-gDNA-R4	CACTTAGGCGACTTTCAACTTCC	
Amh-gDNA-F5	TGGAAGTTGAAAGTCGCCTA	1359
Amh-gDNA-R5	GCTGAAAGGTACTCACTACCTAAT	
Amh-gDNA-F6	AACATTGAGATTCAGAGTCATTATT	610
Amh-gDNA-R6	CTGCAACTCGTATGCACG	
Amh-gDNA-F7	ACAGGAGAAAGCCAGTACCGTG	1555
Amh-gDNA-R7	CGAACGAATAAGTGAACCCTGAT	

**Table 5:** The gRNA sequences and common primer for targeting *CmAmh*.

Primer Name	Sequence (5′→3′)	Locus
Amh-gRNA1	TAATACGACTCACTATAGGAAGTTGAAAGTCGCCTAAG**GTTTTTAGAGCTAGAAATAGC**	Exon 3
Amh-gRNA2	TAATACGACTCACTATAGGACAACATTCACCAGAAA**GTTTTAGAGCTAGAAATAGC**	Exon 4
Common reverse	AAAAAAAGCACCGACTCGGT	

Note: Underlined sequences indicate target sites, bold sequences represent the plasmid scaffold.

## Data Availability

All the data related to this project are available from the corresponding author and will be provided on request.

## Supplementary Materials

The following supporting information can be downloaded at: https://www.mdpi.com/article/10.3390/ijms27198588/s1.

## References

[1] Josso N., Picard J.-Y. (2022). Genetics of anti-Müllerian hormone and its signaling pathway. Best Pract. Res. Clin. Endocrinol. Metab..

[2] Nelson S.M., Davis S.R., Kalantaridou S., Lumsden M.A., Panay N., Anderson R.A. (2023). Anti-Müllerian hormone for the diagnosis and prediction of menopause: A systematic review. Hum. Reprod. Update.

[3] Russell N., Gilmore A., Roudebush W.E. (2022). Clinical utilities of anti-Müllerian hormone. J. Clin. Med..

[4] Rodriguez K.F., Brown P.R., Amato C.M., Nicol B., Liu C.-F., Xu X., Yao H.H.-C. (2022). Somatic cell fate maintenance in mouse fetal testes via autocrine/paracrine action of AMH and activin B. Nat. Commun..

[5] Hayes E., Kushnir V., Ma X., Biswas A., Prizant H., Gleicher N., Sen A. (2016). Intra-cellular mechanism of anti-Müllerian hormone (AMH) in regulation of follicular development. Mol. Cell. Endocrinol..

[6] di Clemente N., Racine C., Pierre A., Taieb J. (2021). Anti-Müllerian hormone in female reproduction. Endocr. Rev..

[7] Kitano J., Ansai S., Takehana Y., Yamamoto Y. (2024). Diversity and convergence of sex-determination mechanisms in teleost fish. Annu. Rev. Anim. Biosci..

[8] Zhou L., Wang D., Liu X. (2026). Amh/Amhr2 homologs: The predominant recurrent sex determining genes in teleosts. Water Biol. Secur..

[9] Hattori R.S., Murai Y., Oura M., Masuda S., Majhi S.K., Sakamoto T., Fernandino J.I., Somoza G.M., Yokota M., Strüssmann C.A. (2012). A Y-linked anti-Müllerian hormone duplication takes over a critical role in sex determination. Proc. Natl. Acad. Sci. USA.

[10] Yamamoto Y., Zhang Y., Sarida M., Hattori R.S., Strüssmann C.A. (2014). Coexistence of genotypic and temperature-dependent sex determination in Pejerrey *Odontesthes bonariensis*. PLoS ONE.

[11] Bej D.K., Miyoshi K., Hattori R.S., Strüssmann C.A., Yamamoto Y. (2017). A duplicated, truncated *Amh* gene is involved in male sex determination in an old world silverside. G3 Genes Genomes Genet..

[12] Li M., Sun Y., Zhao J., Shi H., Zeng S., Ye K., Jiang D., Zhou L., Sun L., Tao W. (2015). A tandem duplicate of anti-Müllerian hormone with a missense SNP on the Y chromosome is essential for male sex determination in Nile tilapia, *Oreochromis niloticus*. PLoS Genet..

[13] Han C., Zhu Q., Lu H., Wang C., Zhou X., Peng C., Tang L., Han L., Chen J., Li S. (2020). Screening and characterization of sex-specific markers developed by a simple NGS method in mandarin fish (*Siniperca chuatsi*). Aquaculture.

[14] Pan Q., Feron R., Yano A., Guyomard R., Jouanno E., Vigouroux E., Wen M., Busnel J.-M., Bobe J., Concordet J.-P. (2019). Identification of the master sex determining gene in northern pike (*Esox lucius*) reveals restricted sex chromosome differentiation. PLoS Genet..

[15] Pan Q., Feron R., Jouanno E., Darras H., Herpin A., Koop B., Rondeau E., Goetz F.W., Larson W.A., Bernatchez L. (2021). The rise and fall of the ancient northern pike master sex-determining gene. eLife.

[16] Peichel C.L., McCann S.R., Ross J.A., Naftaly A.F.S., Urton J.R., Cech J.N., Grimwood J., Schmutz J., Myers R.M., Kingsley D.M. (2020). Assembly of the threespine stickleback Y chromosome reveals convergent signatures of sex chromosome evolution. Genome Biol..

[17] Sardell J.M., Josephson M.P., Dalziel A.C., Peichel C.L., Kirkpatrick M. (2021). Heterogeneous histories of recombination suppression on stickleback sex chromosomes. Mol. Biol. Evol..

[18] Jeffries D.L., Mee J.A., Peichel C.L. (2022). Identification of a candidate sex determination gene in *Culaea inconstans* suggests convergent recruitment of an *Amh* duplicate in two lineages of stickleback. J. Evol. Biol..

[19] Rondeau E.B., Laurie C.V., Johnson S.C., Koop B.F. (2016). A PCR assay detects a male-specific duplicated copy of anti-Müllerian hormone (*Amh*) in the lingcod (*Ophiodon elongatus*). BMC Res. Notes.

[20] Song W., Xie Y., Sun M., Li X., Fitzpatrick C.K., Vaux F., O’Malley K.G., Zhang Q., Qi J., He Y. (2021). A duplicated *Amh* is the master sex-determining gene for *Sebastes* rockfish in the Northwest Pacific. Open Biol..

[21] Hattori R.S., Kumazawa K., Nakamoto M., Nakano Y., Yamaguchi T., Kitano T., Yamamoto E., Fuji K., Sakamoto T. (2022). Y-specific *Amh* allele, *Amhy*, is the master sex-determining gene in Japanese flounder *Paralichthys olivaceus*. Front. Genet..

[22] Ansai S., Montenegro J., Masengi K.W.A., Nagano A.J., Yamahira K., Kitano J. (2022). Diversity of sex chromosomes in Sulawesian medaka fishes. J. Evol. Biol..

[23] Klüver N., Pfennig F., Pala I., Storch K., Schlieder M., Froschauer A., Gutzeit H.O., Schartl M. (2007). Differential expression of anti-Müllerian hormone (*Amh*) and anti-Müllerian hormone receptor type II (*amhrII*) in the teleost medaka. Dev. Dyn..

[24] Miura T., Miura C., Konda Y., Yamauchi K. (2002). Spermatogenesis-preventing substance in Japanese eel. Development.

[25] Luo Q., Ou M., Zhao J., Liu H., Gao D., Wu Y., Zhang L., Chen K. (2020). Expression profile and estrogenic regulation of *Amh* during gonadal sex differentiation in northern snakehead (*Channa argus*). Genes Genom..

[26] Oliveira M.A., Martinez E.R.M., Butzge A.J., Doretto L.B., Ricci J.M.B., Rodrigues M.S., Vigoya A.A.A., Gómez-González N.E., Stewart A.B., Nóbrega R.H. (2021). Molecular characterization and expression analysis of anti-Müllerian hormone in common carp (*Cyprinus carpio*) adult testes. Gene Expr. Patterns.

[27] Liu S., Han C., Huang J., Li M., Yang J., Li G., Lin H., Li S., Zhang Y. (2023). Genome-wide identification, evolution and expression of TGF-β signaling pathway members in mandarin fish (*Siniperca chuatsi*). Int. J. Biol. Macromol..

[28] Wu G.-C., Chiu P.-C., Lyu Y.-S., Chang C.-F. (2010). The expression of *Amh* and *Amhr2* is associated with the development of gonadal tissue and sex change in the protandrous black porgy, *Acanthopagrus schlegeli*. Biol. Reprod..

[29] Hu Q., Guo W., Gao Y., Tang R., Li D. (2015). Molecular cloning and characterization of *Amh* and *Dax1* genes and their expression during sex inversion in rice-field eel *Monopterus albus*. Sci. Rep..

[30] Han Y., Zhao M., Wang L., Yu Z., Wang J., Yu Q., Xiao L., Lu M., Li S., Zhang Y. (2019). Overexpression of anti-Müllerian hormone gene in vivo affects gonad sex differentiation in undifferentiated orange-spotted groupers (*Epinephelus coioides*). Front. Endocrinol..

[31] Wu G.-C., Li H.-W., Tey W.-G., Lin C.-J., Chang C.-F. (2017). Expression profile of *Amh*/Amh during bi-directional sex change in the protogynous orange-spotted grouper *Epinephelus coioides*. PLoS ONE.

[32] Yang K., Chen X., Bao J., Zhai G., Li X., Bao H., Jin X., He J., Wei H., Gao Y. (2026). Improved masculinization effect induced by letrozole via suppression of the feminization through AMH signaling in mandarin fish (*Siniperca chuatsi*). Aquaculture.

[33] Wang W., Tan S., Yang Y., Zhou T., Xing D., Su B., Wang J., Li S., Shang M., Gao D. (2022). Feminization of channel catfish with 17β-oestradiol involves methylation and expression of a specific set of genes independent of the sex determination region. Epigenetics.

[34] Chen K., Guo Y., Rong Y., Li Z., Jiang M., Li R., Zhang X., Li Y., Wei Y., Song Y. (2026). Hormonally and chemically induced male-to-female sex reversal is independent of Y-chromosome number in common carp. Aquaculture.

[35] He A., Li W., Wang W., Ye K., Gong S., Wang Z. (2020). Analysis of DNA methylation differences in gonads of the large yellow croaker. Gene.

[36] Huang X., Qin Q., Gong K., Wu C., Zhou Y., Chen Q., Feng W., Xing Y., Wang C., Wang Y. (2020). Comparative analyses of the *Sox9a*-*Amh*-*Cyp19a1a* regulatory cascade in autotetraploid fish and its diploid parent. BMC Genet..

[37] Shao C., Li Q., Chen S., Zhang P., Lian J., Hu Q., Sun B., Jin L., Liu S., Wang Z. (2014). Epigenetic modification and inheritance in sexual reversal of fish. Genome Res..

[38] Domingos J.A., Budd A.M., Banh Q.Q., Goldsbury J.A., Zenger K.R., Jerry D.R. (2018). Sex-specific *Dmrt1* and *Cyp19a1* methylation and alternative splicing in gonads of the protandrous hermaphrodite barramundi. PLoS ONE.

[39] Ou M., Zhang X., Zhang Y., Lu Y., Yang Z., Chen K., Liu H., Luo Q., Fei S., Zhao J. (2025). Histological observations and transcriptome analyses reveal the gonadal changes in blotched snakehead (*Channa maculata*) during sex reversal induced by 17β-estradiol. Aquac. Rep..

[40] Zhao J., Ou M., Wang Y., Liu H., Luo Q., Zhu X., Chen B., Chen K. (2021). Breeding of YY super-male of blotched snakehead (*Channa maculata*) and production of all-male hybrid (*Channa argus* ♀ × *C. maculata* ♂). Aquaculture.

[41] Ou M., Chen K., Gao D., Wu Y., Chen Z., Luo Q., Liu H., Zhao J. (2020). Comparative transcriptome analysis on four types of gonadal tissues of blotched snakehead (*Channa maculata*). Comp. Biochem. Physiol. Part D Genom. Proteom..

[42] Han C., Liu S., Peng S., Liu S., Zeng J., Chen J., Lin H., Li C., Li S., Zhang Y. (2025). Assembly and analysis of sinipercidae fish sex chromosomes reveals that a supergene drives sex chromosome origin and turnover. Adv. Biotechnol..

[43] Liu H., Wang Y., Guo Q., Tian X., Ma X., Zhang M., Shi X., Ma W., Zhang J., Xu R. (2025). Identification of *Amh* and its potential signaling pathway involved in the ovary development in the largemouth bass (*Micropterus salmoides*). J. Steroid Biochem. Mol. Biol..

[44] Curzon A.Y., Shirak A., Ron M., Seroussi E. (2023). Master-key regulators of sex determination in fish and other vertebrates—A review. Int. J. Mol. Sci..

[45] Zhang X., Wu Y., Zhang Y., Zhang J., Chu P., Chen K., Liu H., Luo Q., Fei S., Zhao J. (2024). Histological observations and transcriptome analyses reveal the dynamic changes in the gonads of the blotched snakehead (*Channa maculata*) during sex differentiation and gametogenesis. Biol. Sex Differ..

[46] O’Donnell L., Smith L.B., Rebourcet D. (2022). Sertoli cells as key drivers of testis function. Semin. Cell Dev. Biol..

[47] Yamaguchi T., Kitano T. (2023). Amh/Amhr2 signaling causes masculinization by inhibiting estrogen synthesis during gonadal sex differentiation in Japanese flounder (*Paralichthys olivaceus*). Int. J. Mol. Sci..

[48] Liu X., Xiao H., Jie M., Dai S., Wu X., Li M., Wang D. (2020). *Amh* regulate female folliculogenesis and fertility in a dose-dependent manner through *Amhr2* in Nile tilapia. Mol. Cell. Endocrinol..

[49] Durlinger A.L.L., Gruijters M.J.G., Kramer P., Karels B., Ingraham H.A., Nachtigal M.W., Uilenbroek J.T.J., Grootegoed J.A., Themmen A.P.N. (2002). Anti-Müllerian hormone inhibits initiation of primordial follicle growth in the mouse ovary. Endocrinology.

[50] Piferrer F., Wang H. (2023). Front Matter. Epigenetics in Aquaculture.

[51] Ma Y., Budde M.W., Zhu J., Elowitz M.B. (2023). Tuning methylation-dependent silencing dynamics by synthetic modulation of CpG density. ACS Synth. Biol..

[52] Shi H., Gao T., Liu Z., Sun L., Jiang X., Chen L., Wang D. (2017). Blockage of androgen and administration of estrogen induce transdifferentiation of testis into ovary. J. Endocrinol..

[53] Ruan Y., Li X., Zhai G., Lou Q., Jin X., He J., Yin Z. (2024). Estrogen signaling inhibits the expression of anti-Müllerian hormone (*Amh*) and gonadal-soma-derived factor (*Gsdf*) during the critical time of sexual fate determination in zebrafish. Int. J. Mol. Sci..

[54] Takatsu K., Miyaoku K., Roy S.R., Murono Y., Sago T., Itagaki H., Nakamura M., Tokumoto T. (2013). Induction of female-to-male sex change in adult zebrafish by aromatase inhibitor treatment. Sci. Rep..

[55] Paul-Prasanth B., Bhandari R.K., Kobayashi T., Horiguchi R., Kobayashi Y., Nakamoto M., Shibata Y., Sakai F., Nakamura M., Nagahama Y. (2013). Estrogen oversees the maintenance of the female genetic program in terminally differentiated gonochorists. Sci. Rep..

[56] Sun L.-N., Jiang X.-L., Xie Q.-P., Yuan J., Huang B.-F., Tao W.-J., Zhou L.-Y., Nagahama Y., Wang D.-S. (2014). Transdifferentiation of differentiated ovary into functional testis by long-term treatment of aromatase inhibitor in Nile tilapia. Endocrinology.

[57] Lin Q., Mei J., Li Z., Zhang X., Zhou L., Gui J.-F. (2017). Distinct and cooperative roles of *Amh* and *Dmrt1* in self-renewal and differentiation of male germ cells in zebrafish. Genetics.

[58] Ou M., Huang R., Yang C., Gui B., Luo Q., Zhao J., Li Y., Liao L., Zhu Z., Wang Y. (2021). Chromosome-Level Genome Assemblies of *Channa argus* and *Channa maculata* and comparative analysis of their temperature adaptability. GigaScience.

[59] Ou M., Chen K., Gao D., Wu Y., Luo Q., Liu H., Zhao J. (2022). Characterization, expression and CpG methylation analysis of *Dmrt1* and its response to steroid hormone in blotched snakehead (*Channa maculata*). Comp. Biochem. Physiol. B Biochem. Mol. Biol..

[60] Zhang Y., Lu Y., Xu F., Zhang X., Wu Y., Zhao J., Luo Q., Liu H., Chen K., Fei S. (2024). Molecular characterization, expression pattern, DNA methylation and gene disruption of *Figla* in blotched snakehead (*Channa maculata*). Animals.

[61] Ou M., Wang F., Li K., Wu Y., Huang S., Luo Q., Liu H., Zhang X., Fei S., Chen K. (2023). Generation of myostatin gene-edited blotched snakehead (*Channa maculata*) using CRISPR/Cas9 system. Aquaculture.

