# Supplementary material for: The Role of Amh in Gonadal Development and Its Response to Exogenous Hormones in Blotched Snakehead (Channa maculata)
Source: Int J Mol Sci. 2026 Sep 25;27(19):8588. doi: 10.3390/ijms27198588 (PMC13665096; doi:10.3390/ijms27198588)
Supplement: Supplementary file 1 [file ijms-27-08588-s001.zip › Revised figure S1.pdf]

Fig.S1

1 gaacgcgaatgcctctctctctttcgatgggtatgccaattgtccacattcactcgtgttgcaaacatctcaaaaagaatccacagtctaaatcatcaaggacac

106 atgatattcctattttggcagcatgtcccccagtcggtgaaaaggggggaggatataaatcctatcatctggagcttgacccattttaaacaggcgattgagaaag

1 M L A V D V F C Y G V L T L Y L T R L C V A L

211 gtctgatcacacaggcacacatttagacagtgaagt**ATG**TTGGCTGTGGATGTCTTCTGCTACGGAGTATTGACGCTCTATTTGACCCGGCTCTGTGTGGCGCTG

24 H V L H G Q Q L M L D P T M T G D D H T M S S T N A G D G L Q T K N P

316 CATGTCTTACATGGACAGCAGCTCATGCTGGACCCAACAATGACAGGAGACGACCACACAATGAGCTCCACAAACGCTGGAGATGGTTTGCAGACCAAAAATCCA

59 P S E T R V S H H H I P H N A P C F V D D L F A A L R E G V G S D G E

421 CCATCAGAAACCAGAGTGTCTCATCATCACATCCCTCACAATGCACCATGCTTTGTAGATGACCTATTTGCAGCGCTGCGTGAAGGTGTGGGCAGCGACGGTGAA

94 L T N S S L T L F G I C T A S E N S S S S V L L E L A K E T S R N Q R

526 CTCACAAACAGCAGTTTGACTCTGTTTGGAATCTGCACAGCATCTGAAAATTCATCAAGCTCAGTTTTATTAGAGCTCGCCAAGGAAACAAGCAGAAACCAGAGA

129 N T F K V M H P T A V L L A E E D K S E T L M L A F D L P Q S S L S N

631 AATACATTTAAGGTTATGCATCCAACTGCAGTGCTGTTGGCAGAGGAAGATAAGAGTGAAACACTCATGTTGGCCTTTGACCTCCCACAATCTTCTTTGTCCAAT

164 T K L L L I L E V E S R L S G G D L D V T F T S Q S L Q P N C Q S V C

736 ACAAAACTTTTGCTAATCTTGGAAGTTGAAAGTCGCCTAAGTGGAGGAGACCTGGACGTCACCTTCACCAGTCAGTCGCTGCAGCCTAACTGTCAGTCTGTGTGC

199 I S G E T Q Y I L L T G R I S E D N I H Q K W R I S V K T K S P D M K

841 ATTTCAGGAGAAACACAGTATATACTATTGACAGGAAGAATATCAGAGGACAACATTCACCAGAAATGGAGGATTTCTGTCAAGACAAAATCCCCTGACATGAAA

234 Q S L K Y V L I G G K S G S N I S L P L L L L F Y G E L E T D T R Y T

946 CAAAGCCTAAAGTACGTCCTGATTGGTGGGAAATCAGGAAGCAACATTAGCTTGCCTCTACTTCTGCTTTTCTATGGGGAACTAGAAACTGATACAAGATACACA

269 H F S G S S Q A S S R T F S F L C E L K R F L G D I L P Q E P P R S P

1051 CATTTTTCAGGCTCATCTCAGGCCTCCTCCAGGACATTCTCCTTCCTTTGTGAGCTGAAACGGTTCCTAGGTGATATTCTGCCCCAGGAACCCCCTCGATCCCCT

304 P L Q L D S L Q S L P P L A I G L S S S E I L L A G L I N S S A P T I

1156 CCTCTCCAGCTGGACTCCTTACAGTCCCTGCCTCCTCTGGCAATCGGCTTATCCTCCAGCGAGATCCTGCTGGCAGGACTGATCAACTCCTCAGCCCCCACTATC

339 F S F T S R G S M F Q V H H G E L A L S P T L L D E L R E R L Q Q T V

1261 TTTTCCTTCACCAGCAGGGGCTCCATGTTTCAGGTGCATCATGGAGAGTTGGCCCTGTCTCCTACACTGTTGGACGAGCTCAGGGAGAGGTTACAGCAGACTGTG

374 V Q I Q H V I R E E D I G Y R A I E R L G R L K A L S V F P K K E P A

1366 GTGCAGATACAGCATGTAATAAGGGAAGAGGACATAGGTTACAGAGCCATAGAGAGGCTGGGGAGACTCAAAGCCCTCAGTGTGTTTCCGAAGAAGGAACCAGCA

409 A G E S Q Y R A F L L L K A L Q T V A R A Y E L Q R G L R A T R A D P

1471 GCAGGAGAAAGCCAGTACCGTGCTTTTCTTCTGCTTAAGGCTCTGCAGACTGTGGCCCGTGCATACGAGTTGCAGAGAGGTCTGCGTGCCACCAGAGCAGACCCC

444 N N P V R S N S C G L R G L T V S L E K H V V G P N N A N I N N C H G

1576 AACAATCCAGTGAGAAGCAACAGCTGTGGGCTGAGGGGCCTCACTGTGTCCCTTGAAAAGCACGTTGTGGGTCCAAACAATGCCAACATTAACAACTGTCATGGC

479 S C T F P L V N S N N H A V L L N S H I E N T E N V D E R A P C C V P

1681 TCTTGTACATTCCCTCTGGTCAACAGCAACAACCACGCTGTTCTGCTCAACTCCCACATTGAAAACACAGAAAATGTGGATGAGCGGGCTCCCTGCTGTGTGCCT

514 V A Y E A L E V M D F N E H G T Y L S I K P D M V A M Q C G C R *

1786 GTGGCCTATGAAGCCCTGGAGGTGATGGATTTCAATGAGCATGGTACTTACCTCTCTATTAAACCAGATATGGTGGCAATGCAATGTGGATGCCGC**TGA**aggtct

1891 tttcttttgtatttctggcaccaaggccatttcacttgtctggtttaacagccgttgatagtagtttgtagtatctcttgctgtcactaattatatttattcaca

1996 cagcattattacactggcacacatattaatactcacaggcctatttagtatacacctatttgtagtagatgaatgcaacagaatggtttttacacataatcatgg

2101 ttattaaaaacttaaatattatatttcagatttgaattcagatgtcagtattttcatctccatctgaataaactatacaattttaattgtttacaagatgttatt

2206 taaataataattgtttaacctcattacaaatattttaaaacccaaaaagatagatttgaccaagccatttggtcatttagtgataaatctgaagacctcctcagc

2311 tagttaaatatggggtaaatgtaacattctctgtatttatatgaataaattcaagttatactcaaaaaaaaaaaaaaaaaaaaaaaaaa
