# Supplementary material for: The Role of Amh in Gonadal Development and Its Response to Exogenous Hormones in Blotched Snakehead (Channa maculata)
Source: Int J Mol Sci. 2026 Sep 25;27(19):8588. doi: 10.3390/ijms27198588 (PMC13665096; doi:10.3390/ijms27198588)
Supplement: Supplementary file 1 [file ijms-27-08588-s001.zip › Revised figure S4.pdf]

Fig. S4.

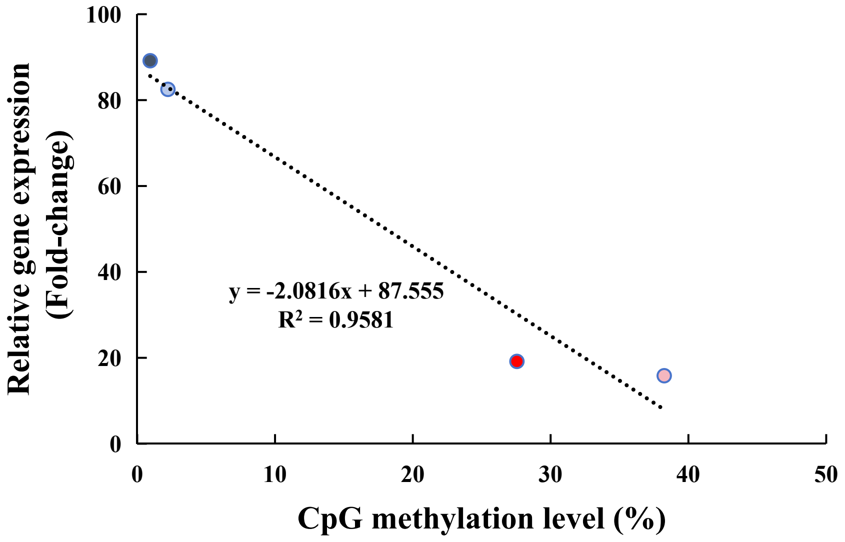

Relative gene expression (Fold-change)
100
80
60
40
20
0
y = -2.0816x + 87.555
R² = 0.9581
0
10
20
30
40
50
CpG methylation level (%)
