# Supplementary material for: The Role of Amh in Gonadal Development and Its Response to Exogenous Hormones in Blotched Snakehead (Channa maculata)
Source: Int J Mol Sci. 2026 Sep 25;27(19):8588. doi: 10.3390/ijms27198588 (PMC13665096; doi:10.3390/ijms27198588)
Supplement: Supplementary file 1 [file ijms-27-08588-s001.zip › Supplement figure_S2.pdf]

Fig.S2

```
-1757  caaacaagccagtacagtgatttatttatttttttaagtcctcaatgaaaatgttagctacactcaaagacattgatgggccacagtatgt
                   C/EBPalp    C/EBPalp        Oct-1
-1665  aggagactaaaatcttaagaagagaaataggagttgtccatagcatttcaattcatatttgttttcccagtctgtaaatatactgaaataac
               Oct-1                     C/EBPbeta                  Sp1             C/EBP
-1573  catgatgagaatttggtacctgccaaaaatcgtcccacaaatacactgttgataacctgttgcagctattagacgatagaaaattcttgtca
            C/EBP                              Ap-1                                  C/EBPalp
-1481  ttcctcactgaagatttgtaatgcacaatagccttggctttatctcttgggatacactgctgctgaacagctcaagagaccttgaccgtctg
                  Oct-1            GATA-1
-1389  caaaacagggctgcagccgtgagaccagcgatgggagtaacactctgtgaaagtattcagatctcatgtctgtgcctgtttttcactttcca
                                                C/EBPalp      Ap-1
-1297  gcataaatgataacacataagttaattaataagaatctctcatcactttgtgttaagttcatccaaatgttgcttggtctttgcatttaatt
         GATA-1         Oct-1          Sp1          Oct-1          C/EBPalp
-1205  gtggtcctgtgccatggagcagctgctcctctggttatttttgaataacactgtcacagcctgaccagtcaacagtgaactgatacaattcaa
         C/EBPalp                         C/EBPbeta    ER     Ap-1
-1113  cacattacaggacaatcagaggcacaacagagggaaatacttgatataaaaataaaagttgtgttacaaagaaattagtcaaattaatttgg
        C/EBPalp                            GATA-1       C/EBPalp
-1021  atccattaacatgaggcctttcgactaaagaaatagttcaatattttggggtattatgtctctttactctcttgctgaagtactaatgcgaa
                                                          Sp1
-929   gatttataccacttgtatgtatgtctaacaactatgaaatttggaaaccagtttgaaacagcctggctcagactaaaggtaaaaaaaaacat
        Oct-1                     C/EBPalp                 C/EBPalp
-837   ctgcctgaccagttcccatcatgttattgcttgtttgtttaggtaaaaaatcctttctttgcagttttgtttttactgcaaactccacaaa
                           Oct-1           C/EBPalp
-745   tgctacataaatagaattaatctagtgcataaccatcagttttctatagattagataaaagcaatatattacattgttatttgccatctttt
                                      C/EBPalp                          Oct-1
-653   aaaacctcctttttctgaacacagccacactaatgctttactaatttccagtcttagtactaagcgaactgtttgctgcctcctgtttata
         ER                    Ap-1          GATA-1                        Sp1
-561   attactgctcagatattgtagtgaaatagatcttgtcagctgacacaggatgtttttcttgtcatcttcacttgtactatctggagtagaagt
              Oct-1                 Ap-1     ER
-469   acaatacagtatatttttggcgtttctttaattttacttcagaaactctgcattgctccattgtttttatgagcaacatttaaacaacaccgc
                   C/EBPalp                                GATA-1          Sp1
-377   tgatattcttggctagggaaacattaacaggtcgataagattacaaggaaaagtgtattctttaatcttgaaacctcccttgaggtttttcc
                                        C/EBPbeta                        Sp1
-285   tcaccttcattaatcaaacgctgtatgtctgaaccaacaaaactgccttttgtttgactttcgaacgcttgtattgtctgagtttaacctgc
-193   ttcctgtaggtgaaaaacaaactcttttcagtacagcaaacatctcaaaaagaatccacagtctaaatcatcaaggacacatgatattccta
                 GATA-1                                     Oct-1
-101   ttttggcagcatgtccccagtcggtgaaaggggaggatataaatcctatcatctggagcttgacccattttaaacaggcgattgagaaa
                         Ap-1              C/EBPalp               GATA-1
-9     ggtctgatcacacaggcacacatttagacagtgaagtATGTTGGCTGTGGATGTCTTCTGCTACGGAGTATTGATGCTCTATTTGACCCGGC
               +1
```
