# Supplementary material for: The Role of Amh in Gonadal Development and Its Response to Exogenous Hormones in Blotched Snakehead (Channa maculata)
Source: Int J Mol Sci. 2026 Sep 25;27(19):8588. doi: 10.3390/ijms27198588 (PMC13665096; doi:10.3390/ijms27198588)
Supplement: Supplementary file 1 [file ijms-27-08588-s001.zip › Supplement figure_S3.pdf]

Fig.S3

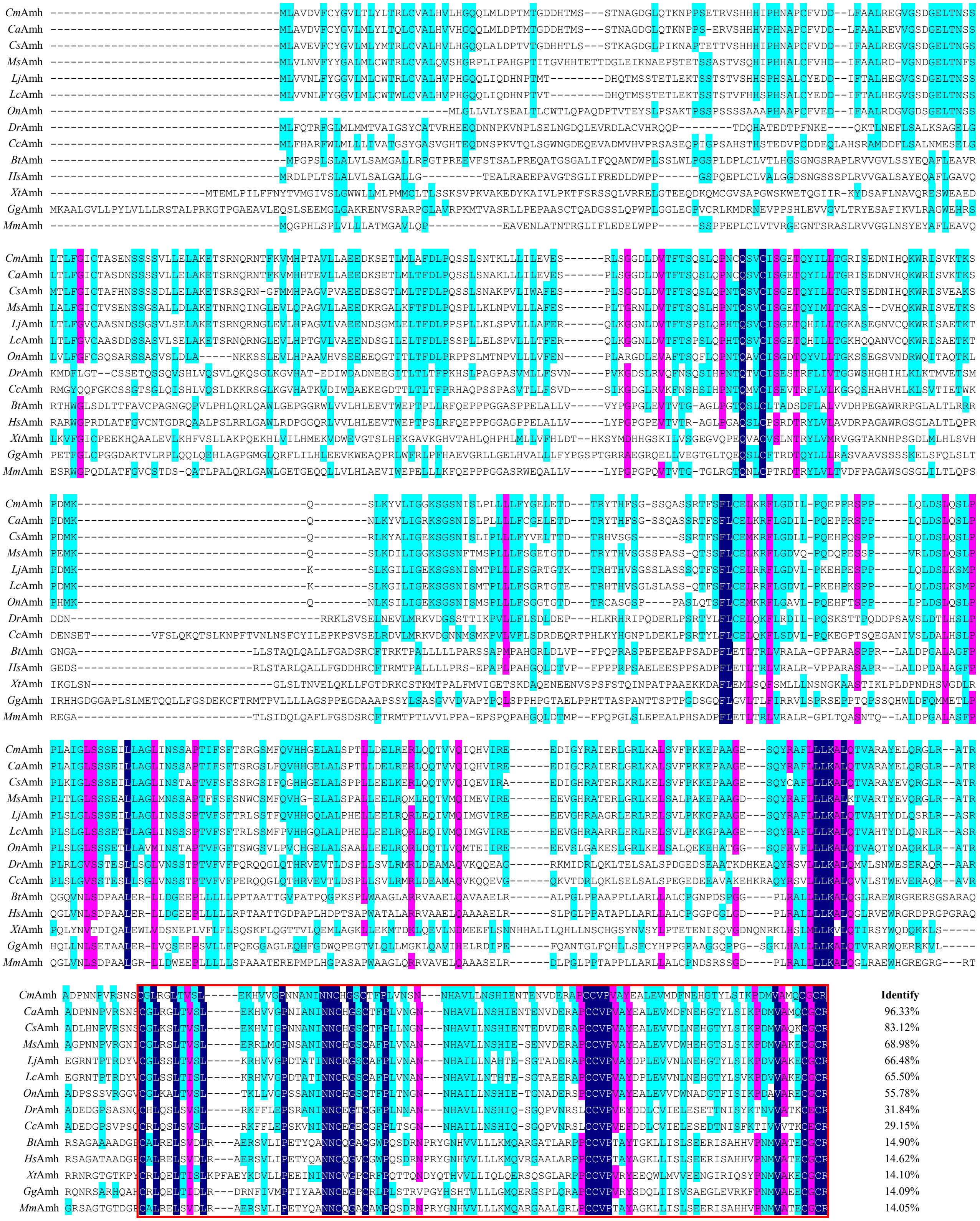
CmAmh
CaAmh
CsAmh
MsAmh
LjAmh
LcAmh
OnAmh
DrAmh
CcAmh
BtAmh
HsAmh
XtAmh
GgAmh
MmAmh
Identify
96.33%
83.12%
68.98%
66.48%
65.50%
55.78%
31.84%
29.15%
14.90%
14.62%
14.10%
14.09%
14.05%
